# Opposite Effects of Apoptotic and Necroptotic Cellular Pathways on Rotavirus Replication

**DOI:** 10.1128/JVI.01222-21

**Published:** 2022-01-12

**Authors:** Mahmoud Soliman, Ja-Young Seo, Yeong-Bin Baek, Jun-Gyu Park, Mun-Il Kang, Kyoung-Oh Cho, Sang-Ik Park

**Affiliations:** a Laboratory of Veterinary Pathology, College of Veterinary Medicine, Chonnam National University, Gwangju, Republic of Korea; b Department of Pathology & Clinical Pathology, Faculty of Veterinary Medicine, Assiut University, Assiut, Egypt; c Jeonnam Bioindustry Foundation Biopharmaceutical Research Center, Hwasun-gun, Jeollanamdo, Republic of Korea; Instituto de Biotecnologia/UNAM

**Keywords:** rotavirus, NSP4, necroptosis, RIPK1/RIPK3/MLKL, apoptosis

## Abstract

Group A rotavirus (RVA), one of the leading pathogens causing severe acute gastroenteritis in children and a wide variety of young animals worldwide, induces apoptosis upon infecting cells. Though RVA-induced apoptosis mediated via the dual modulation of its NSP4 and NSP1 proteins is relatively well studied, the nature and signaling pathway(s) involved in RVA-induced necroptosis are yet to be fully elucidated. Here, we demonstrate the nature of RVA-induced necroptosis, the signaling cascade involved, and correlation with RVA-induced apoptosis. Infection with the bovine NCDV and human DS-1 RVA strains was shown to activate receptor-interacting protein kinase 1 (RIPK1), RIPK3, and mixed-lineage kinase domain-like protein (MLKL), the key necroptosis molecules in virus-infected cells. Using an immunoprecipitation assay, RIPK1 was found to bind phosphorylated RIPK3 (pRIPK3) and pMLKL. pMLKL, the major executioner molecule in the necroptotic pathway, was translocated to the plasma membrane of RVA-infected cells to puncture the cell membrane. Interestingly, transfection of RVA NSP4 also induced necroptosis through the RIPK1/RIPK3/MLKL necroptosis pathway. Blockage of each key necroptosis molecule in the RVA-infected or NSP4-transfected cells resulted in decreased necroptosis but increased cell viability and apoptosis, thereby resulting in decreased viral yields in the RVA-infected cells. In contrast, suppression of RVA-induced apoptosis increased necroptosis and virus yields. Our findings suggest that RVA NSP4 also induces necroptosis via the RIPK1/RIPK3/MLKL necroptosis pathway. Moreover, necroptosis and apoptosis—which have proviral and antiviral effects, respectively—exhibited cross talk in RVA-infected cells. These findings significantly increase our understanding of the nature of RVA-induced necroptosis and the cross talk between RVA-induced necroptosis and apoptosis.

**IMPORTANCE** Viral infection usually culminates in cell death through apoptosis, necroptosis, and, rarely, pyroptosis. Necroptosis is a form of programmed necrosis that is mediated by signaling complexes of the receptor-interacting protein kinase 1 (RIPK1), RIPK3, and mixed-lineage kinase domain-like protein (MLKL). Although apoptosis induction by rotavirus and its NSP4 protein is well known, rotavirus-induced necroptosis is not fully understood. Here, we demonstrate that rotavirus and also its NSP4 protein can induce necroptosis in cultured cells through activation of the RIPK1/RIPK3/MLKL necroptosis pathway. Moreover, rotavirus-induced necroptosis and apoptosis have opposite effects on viral yield, i.e., they function as proviral and antiviral processes, respectively, and counterbalance each other in rotavirus-infected cells. Our findings provide important insights for understanding the nature of rotavirus-induced necroptosis and the development of novel therapeutic strategies against infection with rotavirus and other RNA viruses.

## INTRODUCTION

Programmed cell death (PCD) represents a homeostasis system that controls several aspects of host cell life (1). Different PCD pathways are now being identified based on biochemical features to improve our understanding of cell responses to various stimuli (2). A functional classification of PCD subroutines, including extrinsic apoptosis, caspase-dependent or -independent intrinsic apoptosis, regulated necrosis, autophagic cell death, mitotic catastrophe, anoikis, entosis, parthanatos, pyroptosis, netosis, and cornification has been proposed by the Nomenclature Committee on Cell Death (2). Among them, three forms of cell death, i.e., apoptosis, regulated necrosis (necroptosis), and, rarely, pyroptosis, may be elicited by viral infection (3, 4). Apoptosis is the most extensively studied cell death modality in the context of a wide range of human and animal viral infections (3, 5). Apoptosis can be initiated by either extrinsic signals, such as death cytokines, or intrinsic stimuli (2, 6), resulting in the sequential activation of a family of cysteine-aspartic acid proteases called caspases (2, 5, 6). Both apoptosis pathways can activate caspase-3, the main executioner caspase, following the activation of initiator caspase-8 through the activation of cell surface death receptors in the extrinsic pathway or following the release of proapoptotic factors from the mitochondria and activation of the initiator caspase-9 (2, 5, 6). Recently, caspase-independent intrinsic apoptosis, which can be induced by either the apoptosis-inducing factor, endonuclease G, or high-temperature-requirement protein A2 (2), has been reported for human immunodeficiency virus type 1 (HIV-1)- and influenza virus-infected cells (7, 8).

Necrosis has generally been considered a merely accidental, passive, and uncontrolled cell death (1, 2, 9). However, this view has been challenged in past years by clear evidence indicating that necrosis can occur in a regulated manner and that necrotic cell death plays a prominent role in multiple physiological and pathological settings (1, 2, 10). To differentiate it from passive necrosis, such necrotic cell death is commonly referred to as regulated necrosis, necroptosis, or programmed necrosis (2). Necroptosis can be triggered by death receptors, Toll- and NOD-like receptors, T-cell receptors, genotoxic stress, and pathogens (11), which eventually induce specific signaling cascades (1, 2). The key signaling pathways involved in necroptosis are usually driven by the activation of receptor-interacting protein kinase 1 (RIPK1) (1, 9, 12, 13). Activated RIPK1 and RIPK3 form a complex known as the necrosome through the binding of their RIPK homotypic interaction motif (RHIM) and then phosphorylate each other (1, 9). Next, the phosphorylated RIPK3 (pRIPK3) allows the binding and phosphorylation of mixed-lineage kinase domain-like protein (pMLKL), which eventually translocates to the plasma membrane, where its accumulation ultimately leads to membrane lysis (14–16). This process is known as RIPK1-dependent necroptosis (2, 11). In addition, RIPK3 can also form complexes with DNA-dependent activator of interferon (IFN) regulatory factor (IRF) and the adaptor molecule TIR-domain-containing adaptor-inducing IFN-β (an adaptor protein downstream of Toll-like receptor 3 [TLR3]), thereby inducing RIPK3-dependent necroptosis (2, 11, 17).

Group A rotaviruses (RVAs), which are species of the *Rotavirus* genus in the *Reoviridae* family, are triple-layered nonenveloped viruses with a genome comprising 11 double-stranded RNA (dsRNA) segments encoding six nonstructural and six structural proteins (18, 19). RVAs are the major etiological agent of severe acute gastroenteritis in children and a wide variety of animals worldwide (18, 19). RVA infections were responsible for approximately 215,000 and 108,500 deaths in 2013 and 2016, respectively, in children under the age of 5 years (20–22). RVA infection is mainly restricted to mature enterocytes at the top of the villi of the small intestine in mammalian species and results in epithelial loss and eventual villous atrophy (18, 19). In RVA infections, diarrhea is associated with malabsorption following the destruction of the epithelium (19), the action of the RVA enterotoxin NSP4 (23–25), villus ischemia (26), and the activation of the enteric nervous system (27). In addition, the nausea and vomiting observed in patients with RVA infection are attributed to the release of serotonin from RVA-infected enterochromaffin cells, which, in turn, activates brain structures responsible for causing nausea and vomiting (28, 29).

As morphological characteristics indicative of apoptosis, such as peripheral condensation of chromatin and fragmentation of the nuclei, were initially reported during RVA infection (30), apoptosis has been the most extensively studied cell death modality in response to RVA infection. Apoptosis in RVA-infected cells is modulated by NSP4 and NSP1 during the early and late stages of infection. RVA-induced apoptosis depends on the multiplicity of infection (MOI) and prevails at the late stages of infection (31). NSP4 creates a transmembrane aqueous pore in the endoplasmic reticulum (ER) by virtue of its viroporin domain, consequently increasing the cytoplasmic Ca^2+^ concentration (25, 32) and leading to apoptosis (31, 33). The expression of the proapoptotic protein Bax, which is observed during RVA infection (34), also increases in NSP4-transfected cells, leading to the induction of caspase-dependent intrinsic apoptosis (33). Moreover, NSP4 translocates into the mitochondria and depolarizes them through its interaction with mitochondrial voltage-dependent anion channel and adenine nucleotide translocase proteins, a phenomenon that, in turn, induces caspase-dependent intrinsic apoptosis via the release of cytochrome *c* into the cytosol (31, 33). In contrast, during the initial stage of infection, NSP1 is known to degrade IRF-3, -5, and -7 (35–37) and retinoic acid-inducible gene I (RIG-1) (38), thereby inhibiting RVA-induced apoptosis via the activation of the prosurvival phosphatidylinositol 3-kinase (PI3K)/Akt and NF-κB pathways (39). Moreover, NSP1 interacts with the DNA binding domain of the proapoptotic protein p53 to facilitate its ubiquitination and proteasomal degradation during the initial stage of infection, creating an antiapoptotic environment in RVA-infected cells; however, the extent of this interaction decreases during late infection, leading to the activation of the p53-regulated protein, i.e., Bax, and PUMA to enable the induction of apoptosis by NSP4 (40).

Necroptosis can be induced by multiple enveloped and nonenveloped DNA and RNA viruses (members of at least 11 families) (41–52). In parallel with time-dependent apoptosis, necrosis is induced in a time-dependent manner in response to RVA infection (53). However, whether the necrosis observed during RVA infection is merely accidental, passive, and uncontrolled or regulated is largely unknown, and the exact mechanism by which RVA induces necrosis remains poorly understood. In this study, we examined a potential necroptosis pathway in RVA-infected cells, its role in RVA replication, and its correlation with RVA-induced apoptosis.

## RESULTS

### Induction of necroptosis and apoptosis during RVA infection.

**FIG 1:**
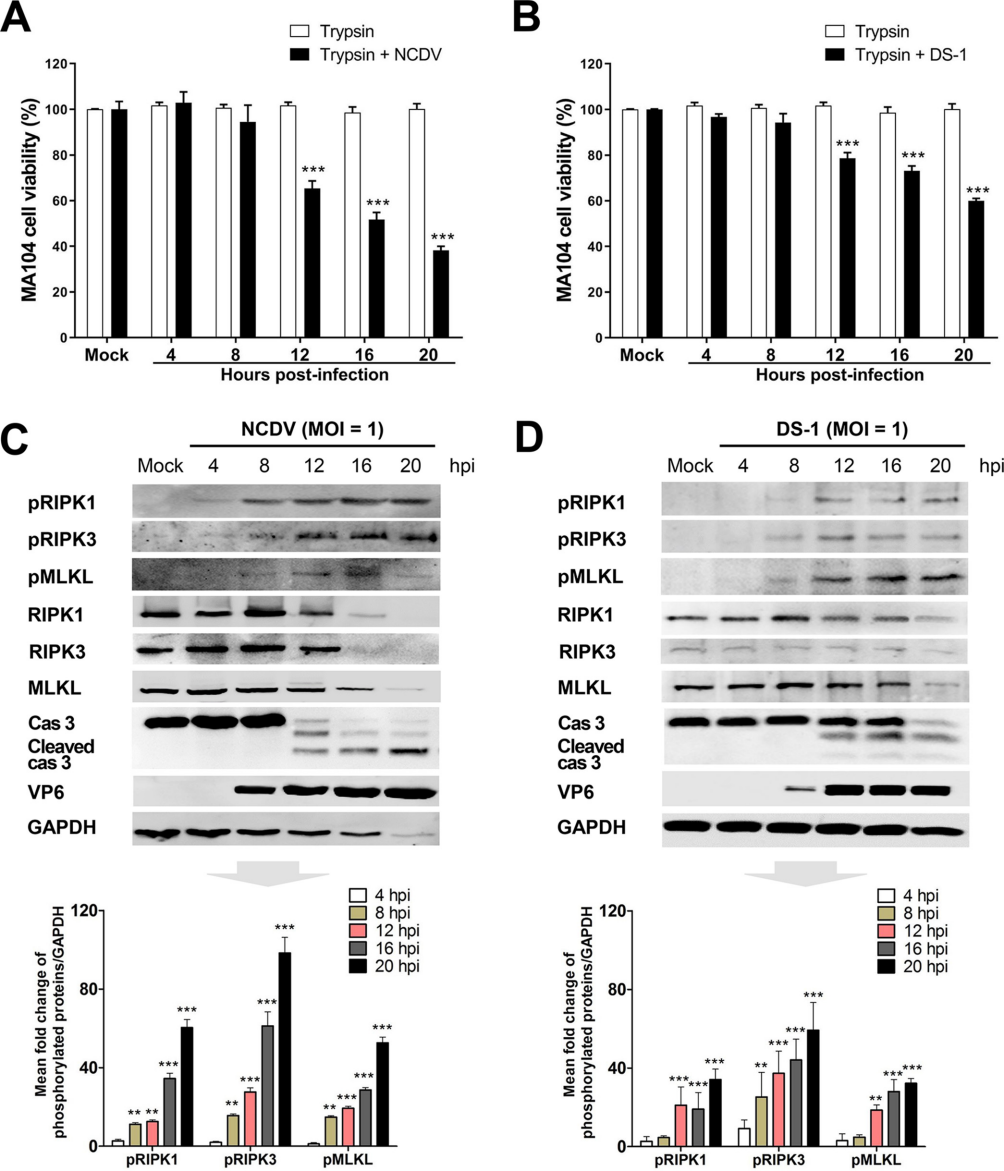
RVA-induced activation of key necroptosis-related molecules in MA104 cells. (A and B) The viability of MA104 cells treated with trypsin or infected with trypsin-activated RVA strain NCDV (A) or DS-1 (B) (MOI = 1) at the indicated time points was evaluated using the MTT assay on an ELISA reader (optical density at 570 nm [OD_570_]). The results are expressed as the mean percentages of viable cells for three independent experiments. (C and D) MA104 cells were treated with trypsin or infected with trypsin-activated RVA strain NCDV (C) or DS-1 (D) (MOI = 1) for the indicated times. The cell lysates were subjected to Western blotting to evaluate the expression of the indicated proteins. GAPDH was used as the loading control. All experiments were performed in triplicate. The relative expression of phosphorylated receptor-interacting protein kinase 1 (pRIPK1), pRIPK3, and mixed-lineage kinase domain-like protein (pMLKL) in virus-infected cells was determined via densitometric analysis and is shown in graphs below each Western blot. The data in panels A to D represent the means ± standard errors of the means from three independent experiments. Differences were evaluated using one-way ANOVA. **, *P < *0.001; ***, *P < *0.0001.

**FIG 2:**
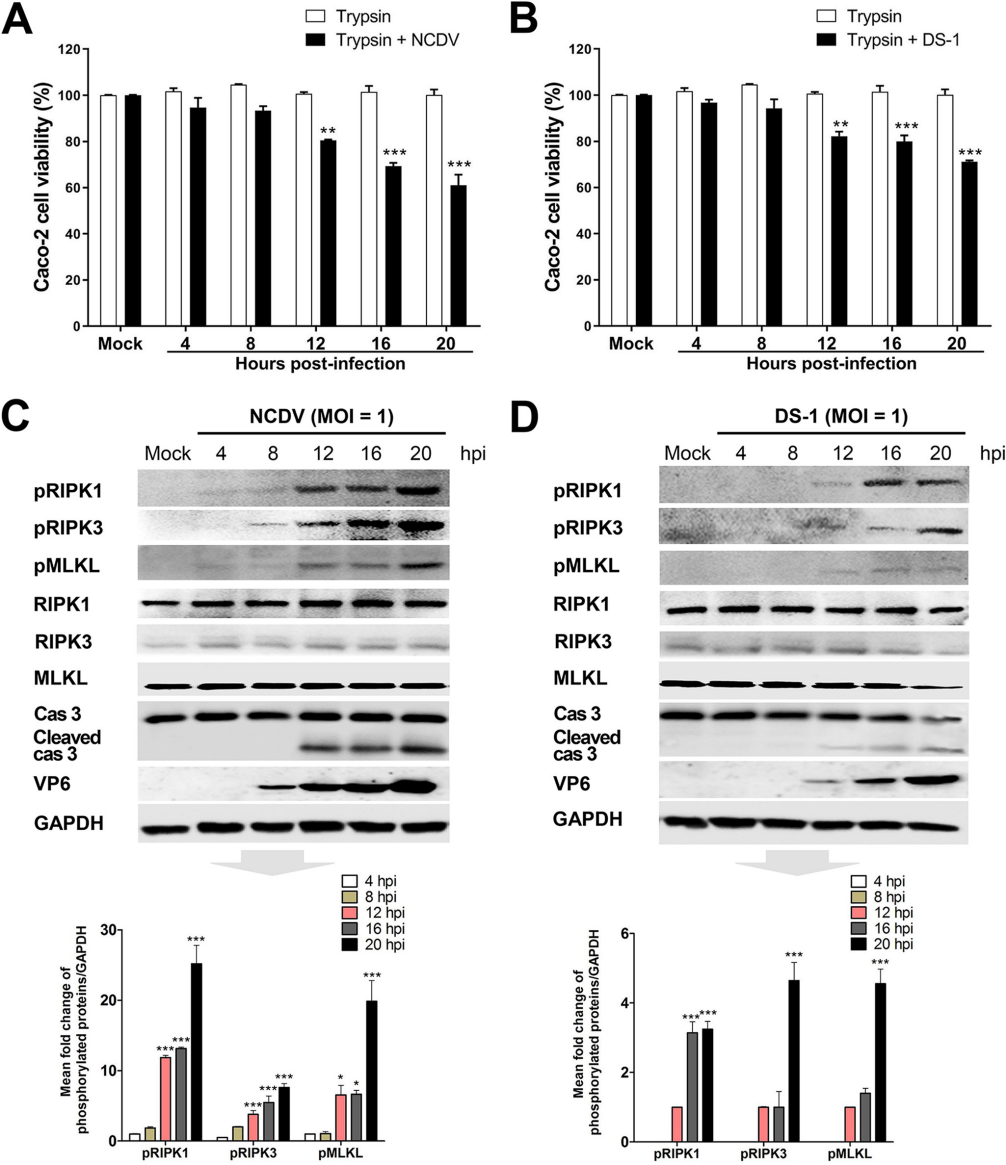
RVA-induced activation of key necroptosis-related molecules in Caco-2 cells. (A and B) The viability of Caco-2 cells treated with trypsin or infected with trypsin-activated RVA strain NCDV (A) or DS-1 (B) (MOI = 1) at the indicated time points was evaluated using the MTT assay on an ELISA reader (OD_570_). The results are expressed as the mean percentages of viable cells for three independent experiments. (C and D) Caco-2 cells were treated with trypsin or infected with trypsin-activated RVA strain NCDV (C) or DS-1 (D) (MOI = 1) for the indicated times. The cell lysates were subjected to Western blotting to evaluate the expression of the indicated proteins. GAPDH was used as the loading control. All experiments were performed in triplicate. The relative expression of pRIPK1, pRIPK3, and pMLKL in virus-infected cells was determined via densitometric analysis and is shown in graphs below each Western blot. The data in panels A to D represent the means ± standard errors of the means from three independent experiments. Differences were evaluated using one-way ANOVA. *, *P < *0.01; **, *P < *0.001; ***, *P < *0.0001.

To determine the nature of RVA-induced cell death modalities, we first performed the 3-(4,5-dimethyl-2-thiazolyl)-2,5-diphenyl-2H-tetrazolium bromide (MTT) assay to examine the viability of MA104 and Caco-2 cells infected with trypsin-activated bovine strain NCDV or human strain DS-1 (MOI = 1) or incubated with a medium containing just trypsin (negative control). MA104 and Caco-2 cells infected with trypsin-activated RVA strains exhibited a significant decrease in viability at 12 to 20 h postinfection (hpi), while mock-infected cells treated with trypsin remained viable up to 20 h posttreatment (Fig. 1A and B and Fig. 2A and B). Next, we determined whether the cell death observed during the early and late stages of RVA infection was due to apoptosis, necroptosis, or both. MA104 and Caco-2 cells infected with trypsin-activated NCDV or DS-1 were harvested, lysed, and subjected to Western blotting. As a negative control, mock-infected cells of both strains were incubated with a medium containing trypsin for 20 h and subjected to Western blotting. Consistent with previous reports (33, 34, 40), cleaved caspase-3, which is the activated form of the main executioner caspase of the extrinsic and intrinsic apoptotic pathways, was detected from 12 to 20 hpi (Fig. 1C and D and Fig. 2C and D) but was not detected in cells incubated with a medium containing trypsin up to 20 h posttreatment. Interestingly, the levels of the phosphorylated forms of RIPK1, RIPK3, and MLKL, the major signaling molecules in the RIPK1-dependent necroptosis pathway, were observed as early as 4 hpi and continuously increased to 20 hpi in response to infection with both RVA strains (Fig. 1C and D and Fig. 2C and D). However, they were not detected in cells incubated with a medium containing trypsin at any time point. These results suggest that RVA can induce apoptosis as well as necroptosis.

### Direct induction of necroptosis and apoptosis in response to RVA infection.

**FIG 3:**
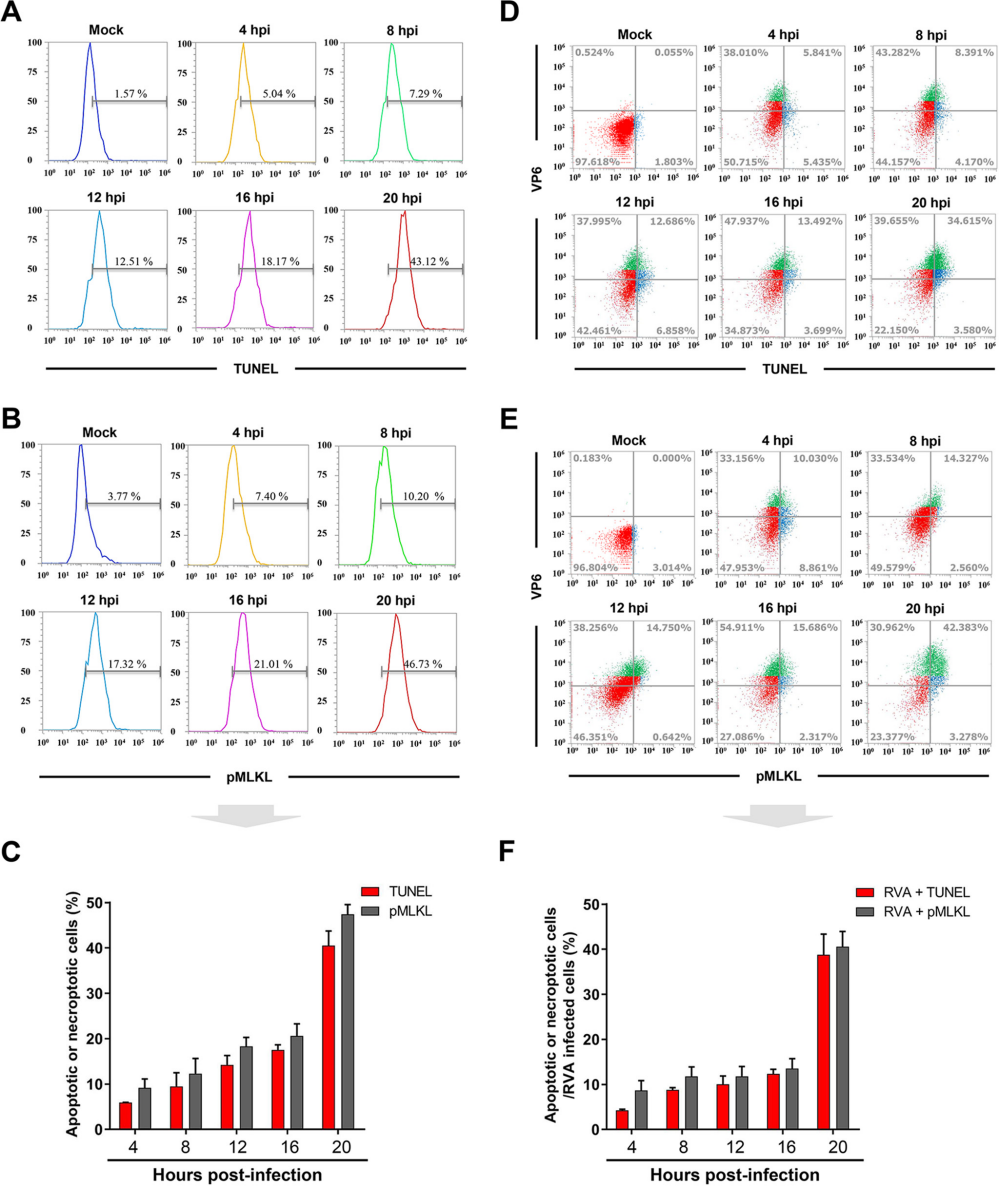
RVA-induced necroptosis and apoptosis. MA104 cells were treated with trypsin or infected with trypsin-activated RVA strain NCDV (MOI = 1) at the indicated time points. (A to C) The cells were harvested at the indicated time points and analyzed using flow cytometry to quantify the number of apoptotic cells using TUNEL staining (A) or necroptotic cells using pMLKL antibody (B) in response to RVA infection. (C) Graphical representation of each apoptotic and necroptotic cell population in response to RVA infection. (D to F) The harvested cells at the indicated time points under the above condition were colabeled for the viral antigen (using VP6 antibody) and either TUNEL or pMLKL antibody and analyzed using flow cytometry to quantify whether RVA-positive cells were apoptotic (D) or necroptotic (E). (F) Graphical representation of RVA VP6-positive and TUNEL-positive apoptotic cells or RVA VP6-positive and pMLKL-positive necroptotic cells at the different time points. Data are shown as the percentage of apoptotic or necrotic cells in RVA-infected cells. The data in panels C and F represent the means ± standard errors of the means from three independent experiments.

**FIG 4:**
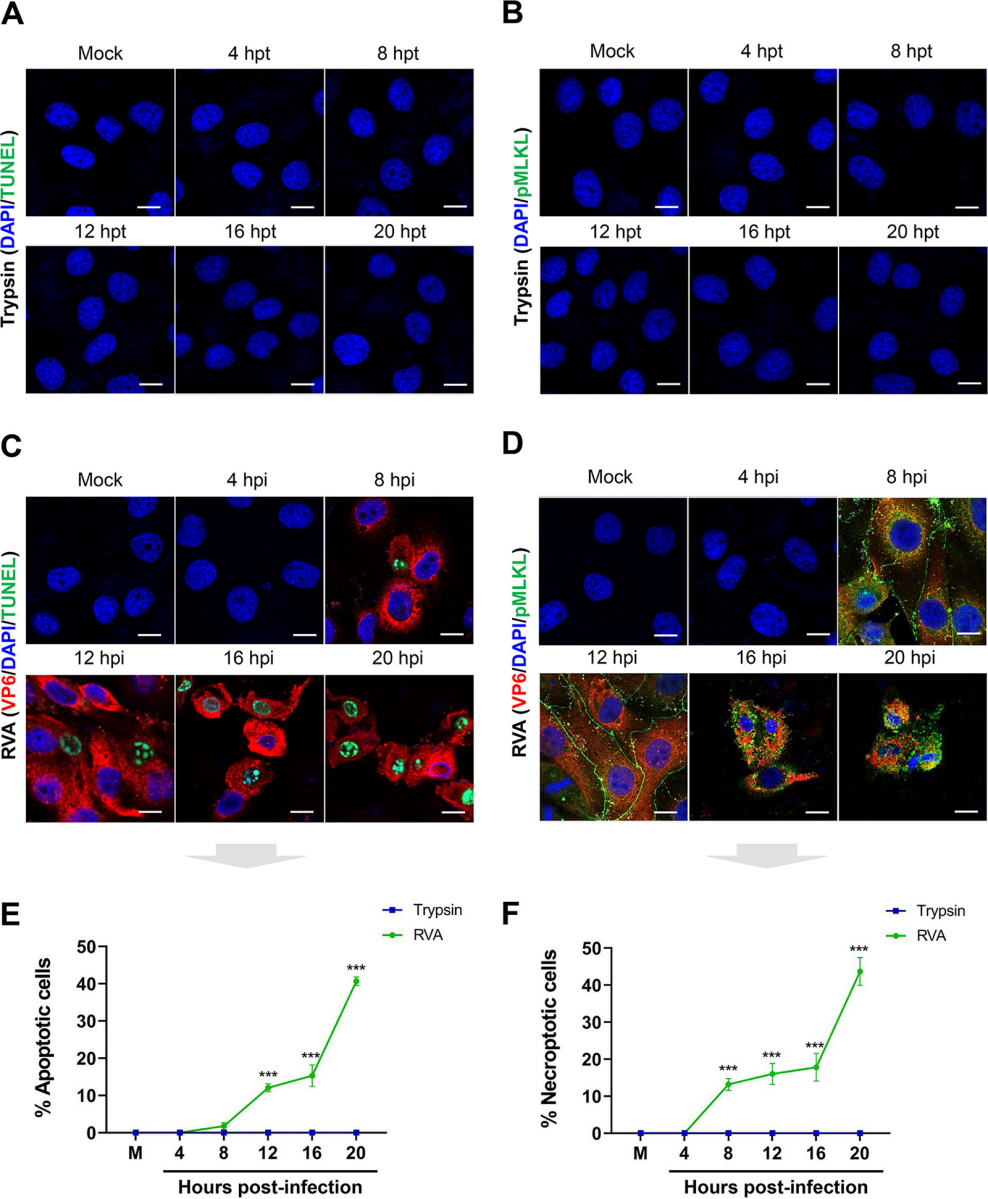
Examination of RVA-induced necroptosis and apoptosis by confocal microscopy. (A and B) MA104 cells treated with trypsin for the indicated times were processed for the TUNEL assay to detect apoptotic cells (A) or for immunofluorescence (IF) to detect necroptotic cells (using an antibody against pMLKL) (B). (C and D) MA104 cells infected with trypsin-activated RVA strain NCDV (MOI = 1) for the indicated times were processed for IF and TUNEL assays to detect the RVA VP6 protein and apoptotic cells (C) and IF to detect the RVA VP6 protein and necroptotic cells (D). The nuclei were stained with DAPI, and all images shown are representative of experiments performed in triplicate. (E and F) The percentages of RVA antigen-positive apoptotic (E) and RVA antigen-positive necroptotic (F) cells were evaluated by counting the cells stained by TUNEL or pMLKL, respectively, as mentioned in Materials and Methods. The data in panels E and F represent the means ± standard errors of the means from three independent experiments. Differences were evaluated using one-way ANOVA. ***, *P < *0.0001. Scale bars = 10 μm.

Apoptotic and necroptotic cells during RVA infection were identified using the terminal deoxynucleotidyltransferase dUTP-biotin nick end-labeling (TUNEL) method and immunofluorescence (IF) with the anti-pMLKL antibody and then quantified using flow cytometry. As shown in Fig. 3A to C, the numbers of apoptotic and necroptotic cells increased in a time-dependent manner in response to RVA infection. Further, the number of cells exhibiting dual positivity for RVA VP6 antigen and apoptotic or necroptotic markers was increased (Fig. 3D to F). To confirm the above results, we used confocal microscopy to visualize apoptotic and necroptotic cells among trypsin-treated and RVA-infected cells. As expected, neither apoptotic nor necroptotic cells were detected in trypsin-treated cells at the indicated times (Fig. 4A and B). However, consistent with previous reports (31, 53), the number of cells positive for the RVA antigen as well as the apoptosis marker increased in a time-dependent manner (Fig. 4C and E). The number of cells positive for the RVA antigen and the necroptosis marker, pMLKL, gradually increased in a time-dependent manner (Fig. 4D to F). pMLKL, the major effector and whose accumulation ultimately leads to membrane lysis (14–16), translocates to the surface of RVA-infected cells (Fig. 4D). These findings indicate that both apoptosis and necroptosis could be directly attributed to RVA infection.

### RVA induces proviral necroptosis via the RIPK1/RIPK3/MLKL pathway.

**FIG 5:**
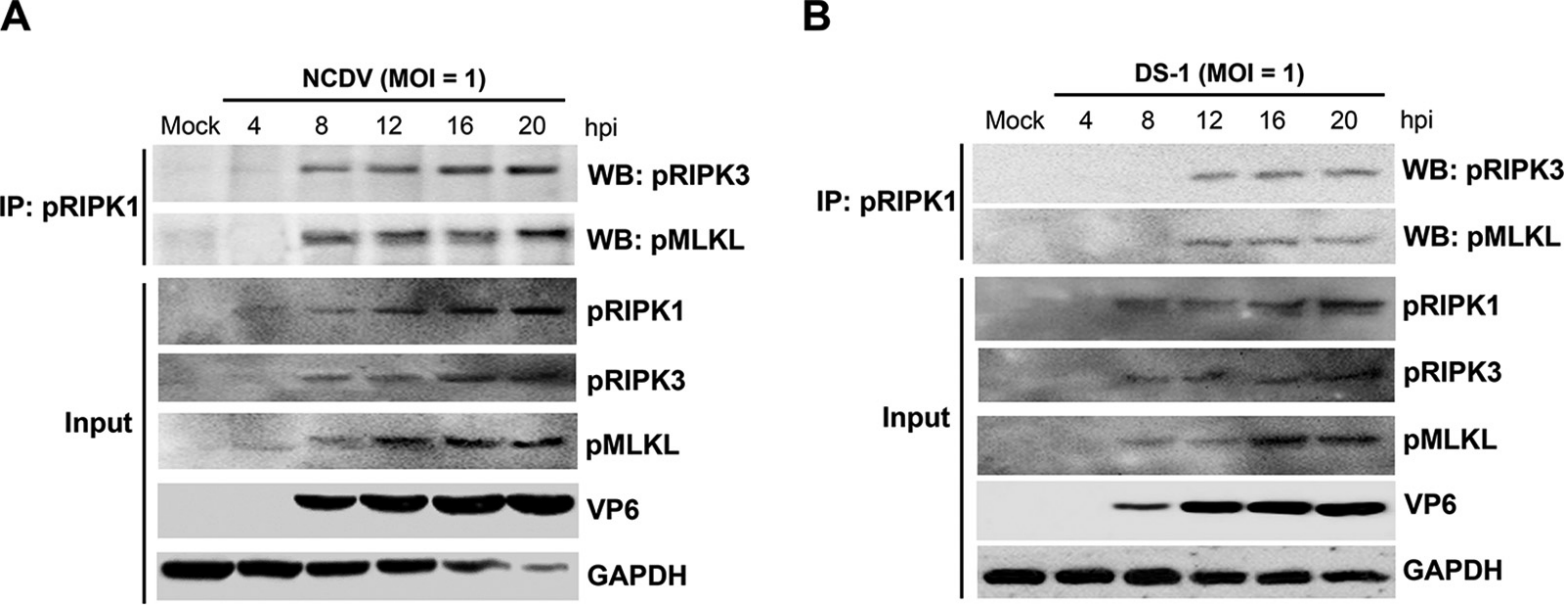
RVA-induced necrosome formation consisting of RIPK1, RIPK3, and MLKL complex. (A and B) MA104 cells were mock infected or infected with RVA strain NCDV (A) or DS-1 (B) (MOI = 1) for the indicated times. The cells were then harvested, and the cell lysates were immunoprecipitated (IP) using an antibody against pRIPK1. Coimmunoprecipitated proteins were analyzed using Western blotting (WB) to detect pRIPK3 and pMLKL using relevant antibodies. All experiments were performed in triplicate, and representative images of different gels from each group are presented.

To identify whether the key molecules involved in necroptosis interact during RVA infection, the MA104 cells were either mock infected or infected with strain NCDV or DS-1 (MOI = 1) for the indicated times, and the cell lysates were immunoprecipitated with an antibody against pRIPK1. As shown in Fig. 5, the pRIPK1 antibody immunoprecipitated pRIPK3 and pMLKL in NCDV- and DS-1-infected cells. These data indicate that RVA-induced necroptosis may occur via the RIPK1-dependent pathway.

**FIG 6:**
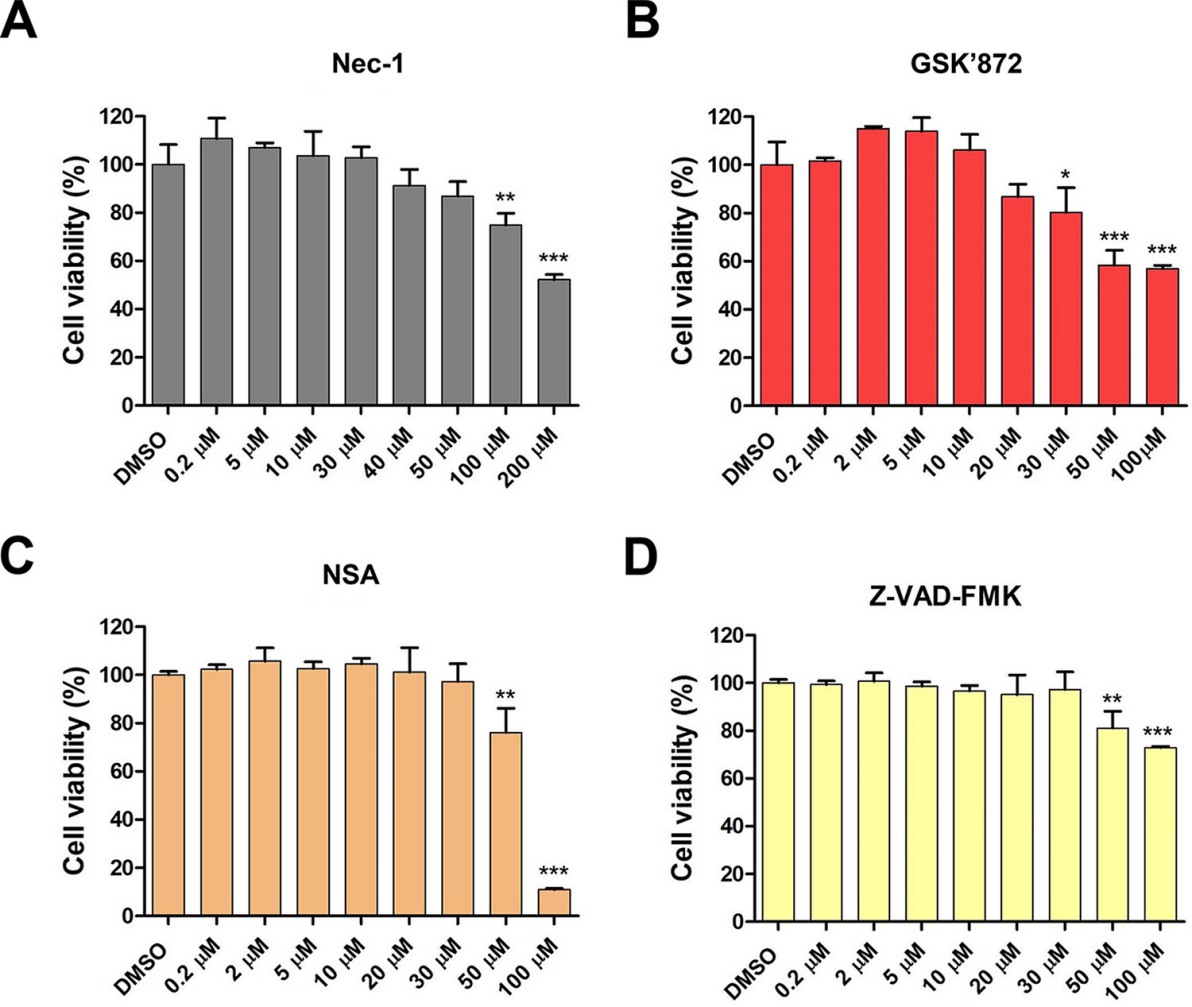
Determination of the cytotoxicity of chemicals using MTT assay. MA104 cells grown in 96-well plates were incubated with various concentrations of the indicated chemicals in triplicate for 24 h at 37°C. Then cell viability was evaluated using the MTT assay on an ELISA reader (OD_570_). The results represent the means ± standard errors of the means from three independent experiments. Differences were evaluated using one-way ANOVA. *, *P < *0.05; **, *P < *0.001; ***, *P < *0.0001.

**FIG 7:**
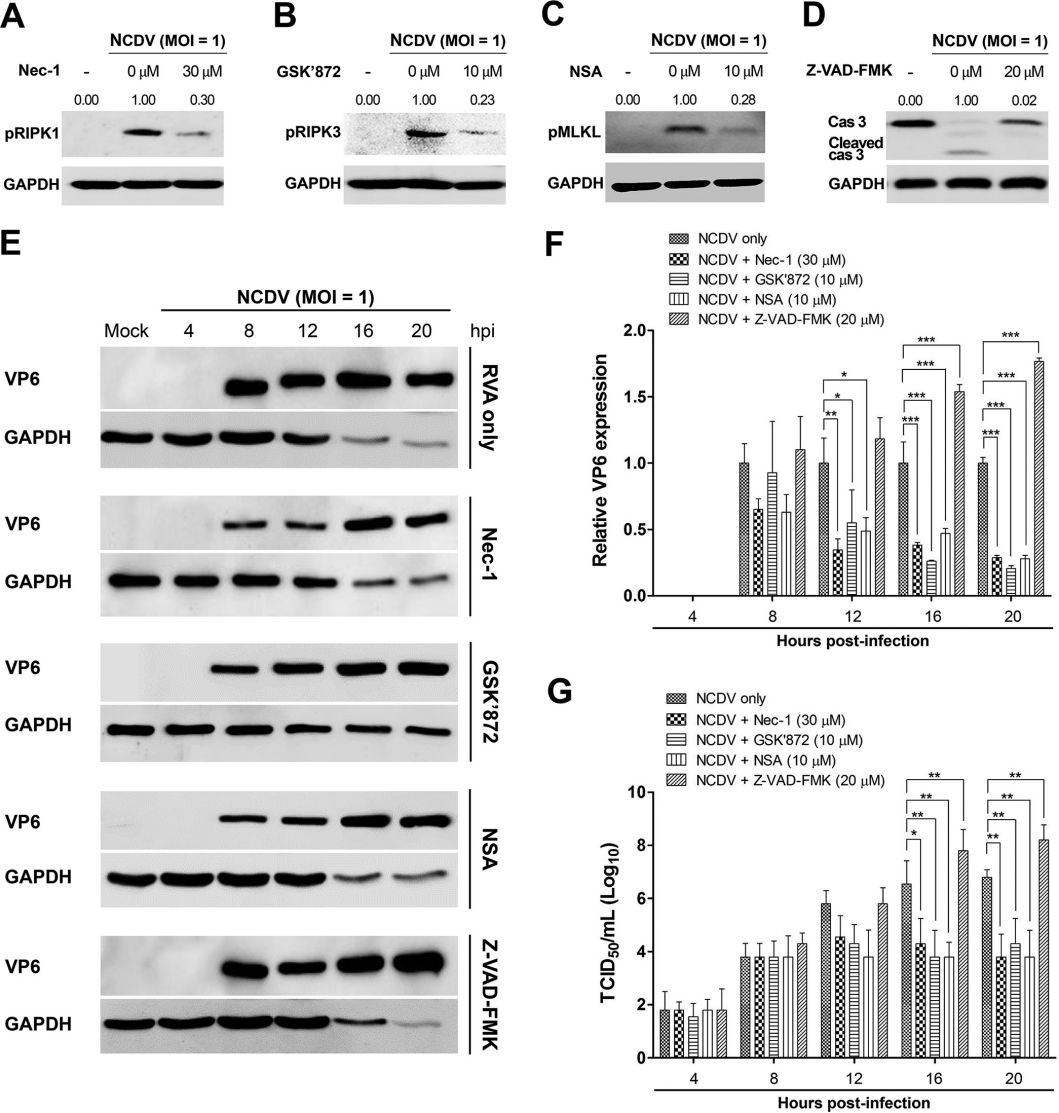
Inhibition of replication of bovine NCDV strain by blocking of necrosome formation in MA104 cells. MA104 cells were infected with bovine RVA strain NCDV (MOI = 1) for 1 h and then incubated with a maintenance medium containing 1 μg/mL trypsin and 30 μM necrostatin-1 (Nec-1; RIPK1 inhibitor), 10 μM GSK’872 (RIPK3 inhibitor), 10 μM necrosulfonamide (NSA; MLKL inhibitor), or 20 μM Z-VAD-FMK (pan-caspase inhibitor). (A to D) The cells harvested at 16 hpi were subjected to Western blotting to determine their target phosphorylated forms of pRIPK1, pRIPK3, pMLKL, and cleaved caspase-3, respectively. GAPDH was used as the loading control. (E) The harvested cells at the indicated time points were subjected to Western blotting to determine RVA VP6 protein using an antibody against the VP6 protein. GAPDH was used as the loading control. (F) Using densitometric analysis, the relative expression of the RVA VP6 protein in cells treated with each necroptosis and apoptosis inhibitor was compared with that in the mock-treated and virus-infected cells. (G) The viral titer under each of the above-described experimental conditions was determined using the median tissue culture infective dose (TCID_50_) assay and compared with that in the mock-treated and virus-infected cells. The data in panels F and G represent the means ± standards error of the means from three independent experiments. Differences were evaluated using one-way ANOVA. *, *P < *0.05; **, *P < *0.001; ***, *P < *0.0001.

**FIG 8:**
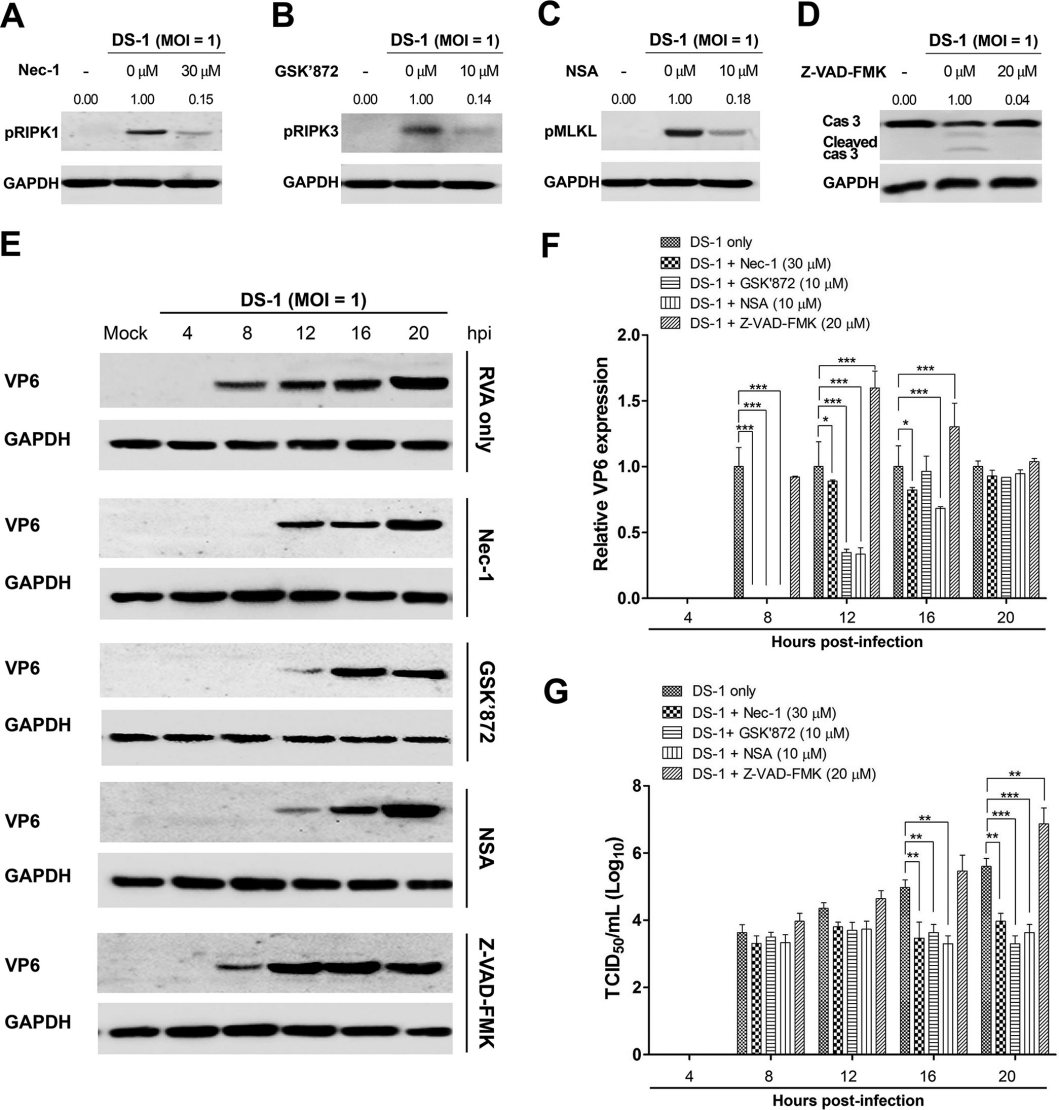
Inhibition of replication of human strain DS-1 by blocking necrosome formation in MA104 cells. MA104 cells were infected with human RVA strain DS-1 (MOI = 1) for 1 h and then incubated with a maintenance medium containing 1 μg/mL trypsin and 30 μM Nec-1 (RIPK1 inhibitor), 10 μM GSK’872 (RIPK3 inhibitor), 10 μM NSA (MLKL inhibitor), or 20 μM Z-VAD-FMK (pan-caspase inhibitor). (A to D) The cells harvested at 16 hpi were subjected to Western blotting to determine their target phosphorylated forms of pRIPK1, pRIPK3, pMLKL, and cleaved caspase-3, respectively. GAPDH was used as the loading control. (E) The harvested cells at the indicated time points were subjected to Western blotting to determine RVA VP6 protein using an antibody against the VP6 protein. GAPDH was used as the loading control. (F) Using densitometric analysis, the relative expression of the RVA VP6 protein in cells treated with each necroptosis and apoptosis inhibitor was compared with that in the mock-treated and virus-infected cells. (G) The viral titer under each of the above-described experimental conditions was determined using the TCID_50_ assay and compared with that in the mock-treated and virus-infected cells. The data in panels F and G represent the means ± standard errors of the means from three independent experiments. Differences were evaluated using one-way ANOVA. *, *P < *0.05; **, *P < *0.001; ***, *P < *0.0001.

**FIG 9:**
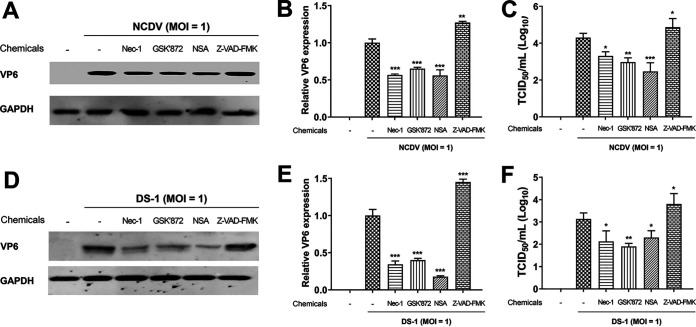
Inhibition of replication of the bovine NCDV and human DS-1 strains by blocking necrosome formation in Caco-2 cells. Caco-2 cells were infected with either bovine RVA NCDV or human RVA DS-1 (MOI = 1) for 1 h and then incubated with a maintenance medium containing 1 μg/mL trypsin and 30 μM Nec-1 (RIPK1 inhibitor), 10 μM GSK’872 (RIPK3 inhibitor), 10 μM NSA (MLKL inhibitor), or 20 μM Z-VAD-FMK (pan-caspase inhibitor). (A and D) The cells harvested at 16 hpi were subjected to Western blotting to determine RVA VP6 protein using an antibody against the VP6 protein. GAPDH was used as the loading control. (B and E) Using densitometric analysis, the relative expression of the RVA VP6 protein in cells treated with each necroptosis and apoptosis inhibitor was compared with that in the mock-treated and virus-infected cells. (C and F). The viral titer under each of the above-described experimental conditions was determined using the TCID_50_ assay and compared with that in the mock-treated and virus-infected cells. The data in panels B, C, E, and F represent the means ± standard error of the mean from three independent experiments. Differences were evaluated using one-way ANOVA. *, *P < *0.05; **, *P < *0.001; ***, *P < *0.0001.

Although necroptosis and apoptosis are generally considered antiviral mechanisms, they are also considered beneficial to the virus in some cases, as they may promote viral release and spread (44, 49, 50, 54, 55). In this study, we used chemical inhibitors specific for each key molecule involved in RIPK1-dependent necroptosis to determine the role of necroptosis in RVA replication. We first determined the optimal nontoxic concentrations of the RIPK1 inhibitor necrostatin-1 (Nec-1), RIPK3 inhibitor GSK’872, MLKL inhibitor necrosulfonamide (NSA), and pan-caspase inhibitor Z-VAD-FMK in the MA104 cells using the MTT assay. We found that Nec-1, GSK’872, NSA, and Z-VAD-FMK were nontoxic to cells at optimal concentrations of 30, 10, 10, and 20 μM, respectively (Fig. 6). We next tested whether blocking of the RIPK1/RIPK3/MLKL signaling pathway by each of the inhibitors influences RVA replication. Treatment of NCDV-infected or DS-1-infected MA104 cells with Nec-1, GSK’872, NSA, and Z-VAD-FMK significantly reduced their phosphorylated forms, pRIPK1, pRIPK3, pMLKL, and cleaved caspase-3, respectively (Fig. 7A to D and Fig. 8A to D). Moreover, treatment with each chemical inhibitor suppressed the expression RVA proteins in both NCDV-infected (Fig. 7E and F) and DS-1-infected (Fig. 8E and F) MA104 cells. RVA titers also decreased significantly at 16 and 20 hpi (Fig. 7G and 8G). In addition, modulation of viral growth with these chemicals was observed in the NCDV- or DS-1-infected Caco-2 cells (Fig. 9).

**FIG 10:**
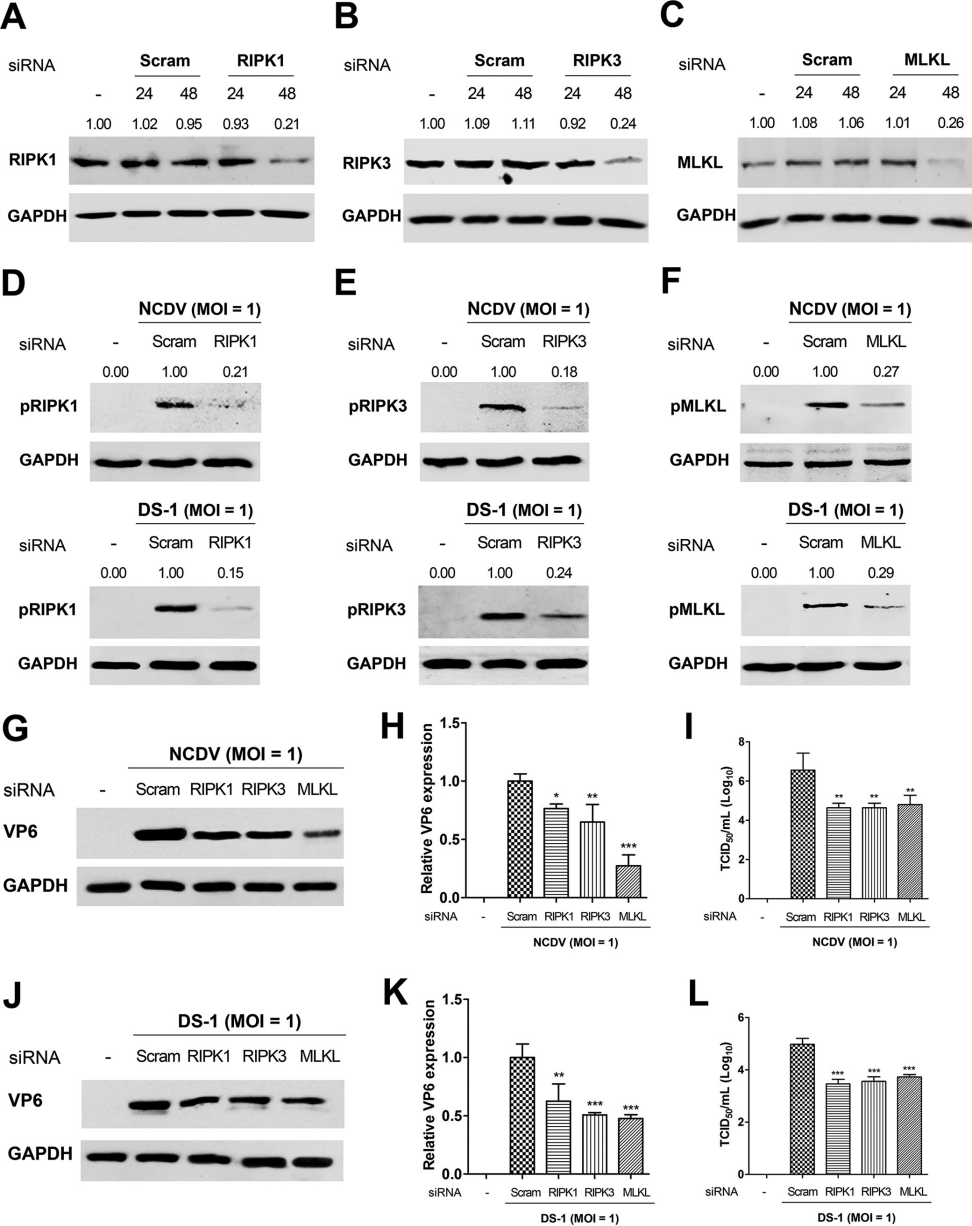
Inhibition of replication of the RVA strains NCDV and DS-1 via the silencing necroptosis molecules. (A to C) Mock-infected MA104 cells were transfected with either scrambled siRNA or siRNAs against RIPK1 (A), RIPK3 (B), or MLKL (C) and harvested at 24 and 48 h posttransfection. Knockdown of RIPK1, RIPK3, or MLKL protein levels was evaluated via Western blot analysis. GAPDH was used as the loading control. (D to F) The transfected cells with either scrambled siRNA or siRNAs against RIPK1, RIPK3, or MLKL were infected with NCDV or DS-1 and harvested at 16 hpi. The cell lysates were subjected to Western blotting to determine their target phosphorylated forms, pRIPK1, pRIPK3, and pMLKL, respectively. GAPDH was used as the loading control. (G to L) MA104 cells were transfected with either scrambled siRNA or siRNAs against RIPK1, RIPK3, or MLKL and incubated for 48 h. Afterward, cells were infected with RVA strain NCDV (G to I) or DS-1 (J to L) at an MOI of 1 and incubated for 16 h. The cells were then processed for Western blotting to check viral protein synthesis (G and J) and relative viral VP6 protein using densitometric analysis (H and K) and for TCID_50_ assay to count progeny virus production (I and L). The data in panels H, I, K, and L were obtained by comparing the results of RVA infection after transfection with the scrambled siRNA and the results of RVA infection after transfection with each siRNA against RIPK1, RIPK3, or MLKL and represent the means ± standard errors of the means from three independent experiments. Differences were evaluated using one-way ANOVA. *, *P < *0.05; **, *P < *0.001; ***, *P < *0.0001.

We further examined the silencing efficacy of small interfering RNAs (siRNAs) against RIPK1, RIPK3, or MLKL on replication of both strains NCDV and DS-1 in the infected cells. The Western blotting results showed that these siRNAs significantly silenced RIPK1, RIPK3, and MLKL in mock-infected MA104 cells (Fig. 10A to C). Moreover, silencing of RIPK1, RIPK3, and MLKL via transfection of each corresponding siRNA in NCDV- or DS-1-infected cells significantly suppressed expression of pRIPK1, pRIPK3, and pMLKL, respectively (Fig. 10D to F). Knockdown of RIPK1, RIPK3, or MLKL in the background of infection with either RVA NCDV or DS-1 suppressed the expression of RVA proteins and titers (Fig. 10G to L). Consistent with the results of a previous report (56), blocking of RVA-induced apoptosis with the pan-caspase inhibitor Z-VAD-FMK increased viral protein expression and titers (Fig. 7 and 9). Taken together, our data suggest that unlike RVA-induced antiviral apoptosis, RVA-induced RIPK1-dependent necroptosis is proviral in nature.

### Counterbalancing of RVA-induced necroptosis and apoptosis.

**FIG 11:**
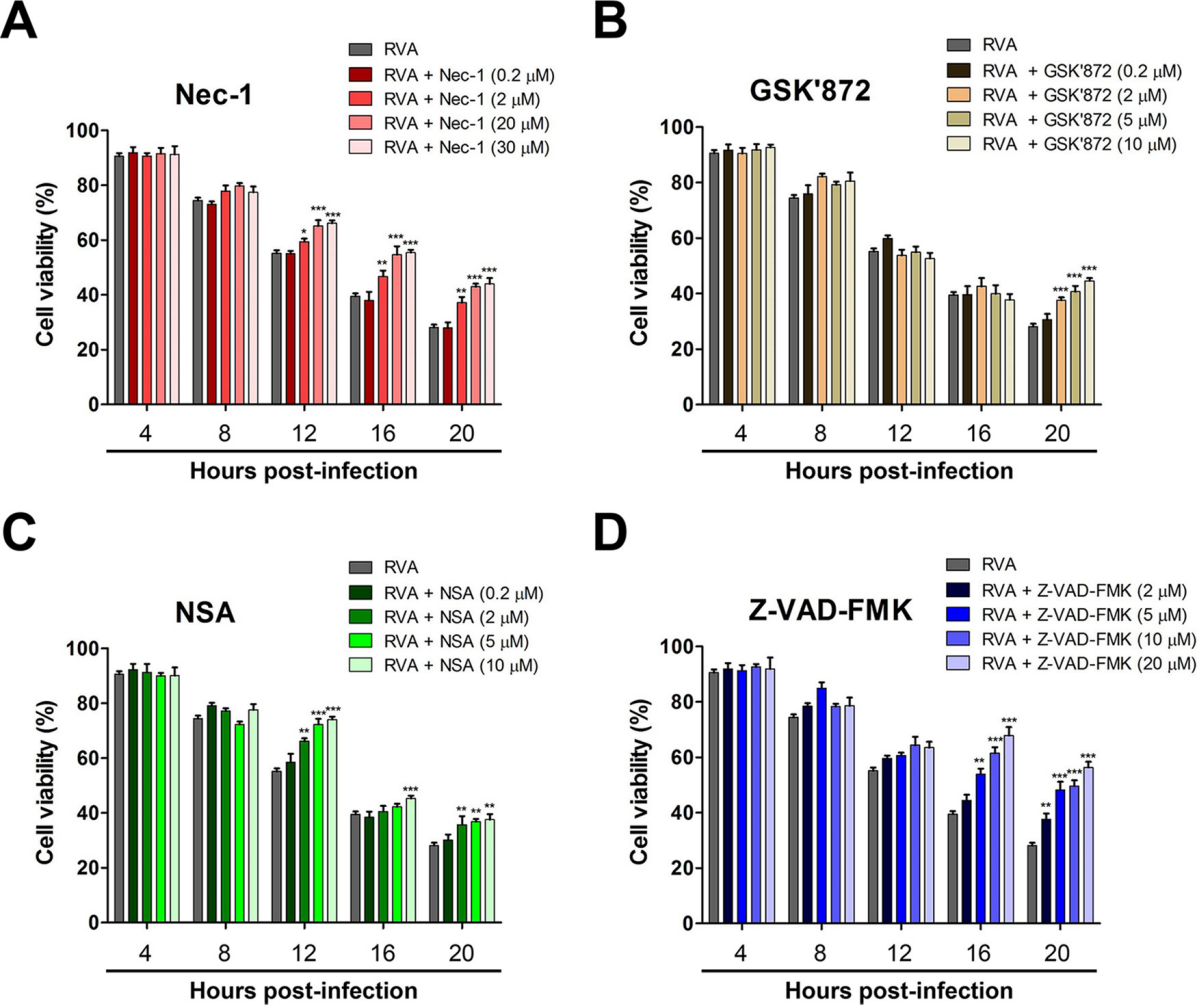
Inhibition of necroptosis or apoptosis reduces RVA-induced cell death. MA104 cells were infected with RVA strain NCDV (MOI = 1) at the indicated time points and posttreated with different concentrations of Nec-1 (A), GSK’872 (B), NSA (C), and Z-VAD-FMK (D) for RIPK1, RIPK3, MLKL, and pan-caspase inhibition. Cell viability at each time point in panels A to D was compared with that in the mock-treated and virus-infected cells. All experiments were performed in triplicate, and the data represent the means ± standard errors of the means. Differences were evaluated using one-way ANOVA. *, *P < *0.05; **, *P < *0.001; ***, *P < *0.0001.

**FIG 12:**
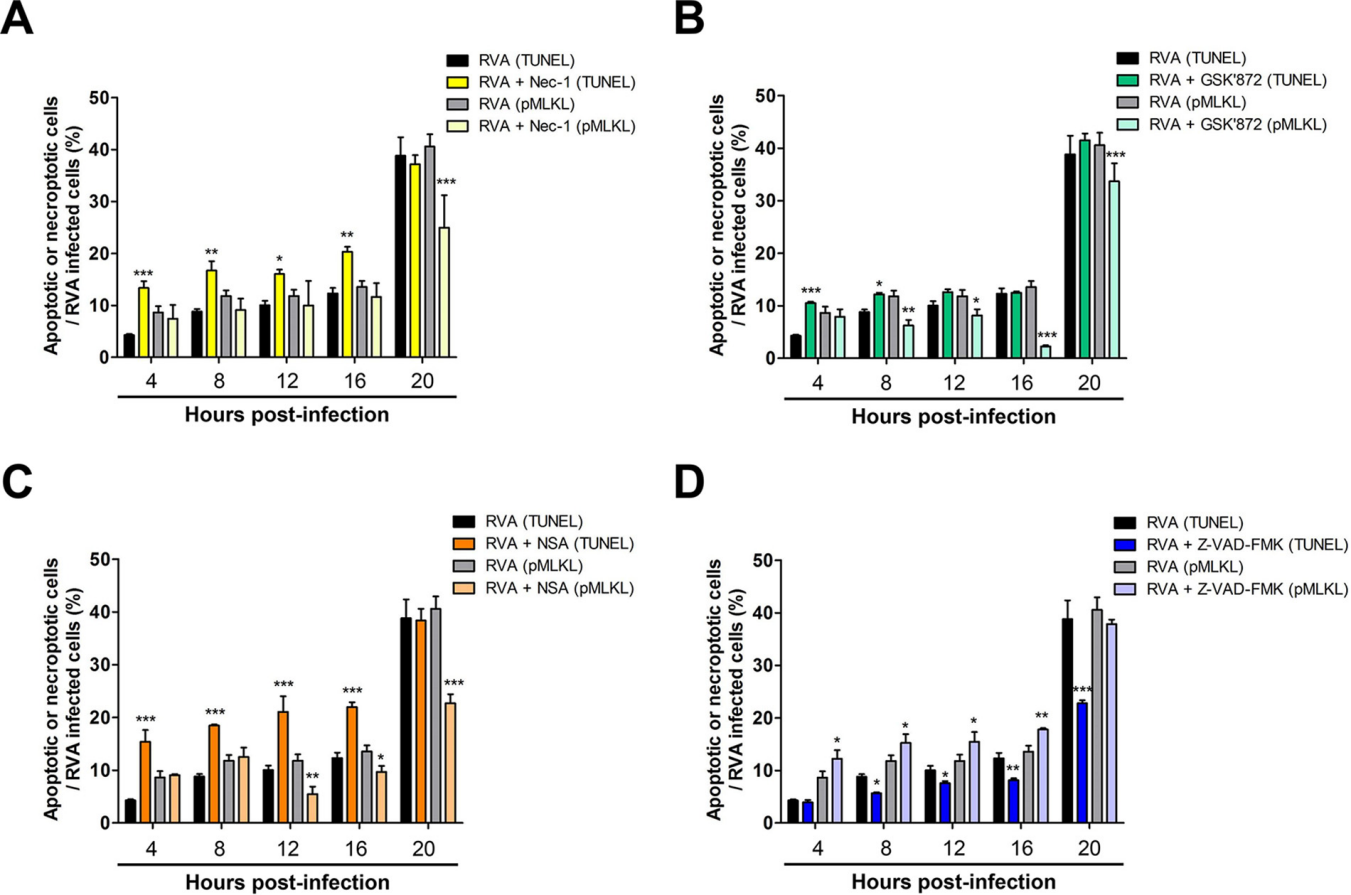
Inhibition of necroptosis converted RVA-induced cell death into apoptosis and vice versa. MA104 cells were infected with RVA strain NCDV (MOI = 1) and then mock treated or treated with 30 μM RIPK1 inhibitor Nec-1 (A), 10 μM RIPK3 inhibitor GSK’872 (B), 10 μM MLKL inhibitor NSA (C), or 20 μM pan-caspase inhibitor Z-VAD-FMK (D). The cells were then harvested at the indicated time points. Double staining was performed for detecting both viral VP6 antigen and necroptosis molecule pMLKL or for detecting both viral VP6 antigen using IF and apoptotic DNA fragments using TUNEL assay and analyzed using flow cytometry to quantify dually positive cells for the RVA VP6 protein with TUNEL-positive signal or pMLKL-positive signal. The data are shown as the percentage of apoptotic or necrotic cells in RVA-infected cells at each time point and compared with those in the mock-treated and virus-infected cells. All experiments were performed in triplicate, and the data represent the means ± standard errors of the means. Differences were evaluated using one-way ANOVA. *, *P < *0.05; **, *P < *0.001; ***, *P < *0.0001.

The transition from apoptosis to necroptosis and vice versa has been reported in response to HIV-1 infection, Theiler’s murine encephalomyelitis virus (TMEV) infection, and tunicamycin stimulation (49, 50, 57). Our results suggested that the decrease in RVA titers in response to the downregulation of the necroptosis molecules, RIPK1, RIPK3, and MLKL, could be attributed to protection from cell death, induction of apoptosis, or both mechanisms. To examine this, we first investigated the viability of RVA-infected cells treated or not with an inhibitor of each molecule. Interestingly, inhibition of RIPK1, RIPK3, and MLKL resulted in an increase in cell viability compared to that of the mock-treated RVA-infected cells, particularly at 12 to 20 hpi, the exception being RIPK3-inhibited cells, which exhibited an increase in viability at 20 hpi (Fig. 11A to C). We further investigated the possible relationship between necroptosis and apoptosis in the context of RVA-induced cell death. Compared to the mock-treated and virus-infected cells, the proportion of apoptotic cells increased after the inhibition of necroptosis in RVA-infected cells by treatment with RIPK1, RIPK3, and MLKL inhibitors, particularly at 4 to 16 hpi (Fig. 12A to C). These results demonstrated that the decreased RVA titers upon the downregulation of RIPK1, RIPK3, or MLKL could be attributed to protection from cell death as well as induction of apoptosis in RVA-infected cells. Moreover, the inhibition of RVA-induced apoptosis by treatment with the pan-caspase inhibitor Z-VAD-FMK resulted in an increase in cell viability at 16 and 20 hpi (Fig. 11D) and increased the proportion of necroptotic cells among RVA-infected cells (Fig. 12D). Taken together, our findings suggest that RVA-induced necroptosis and apoptosis have contrasting functions, acting as proviral and antiviral mechanisms, respectively, and counterbalancing each other in infected cells.

### Induction of apoptosis as well as necroptosis by the NSP4 protein of RVA.

**FIG 13:**
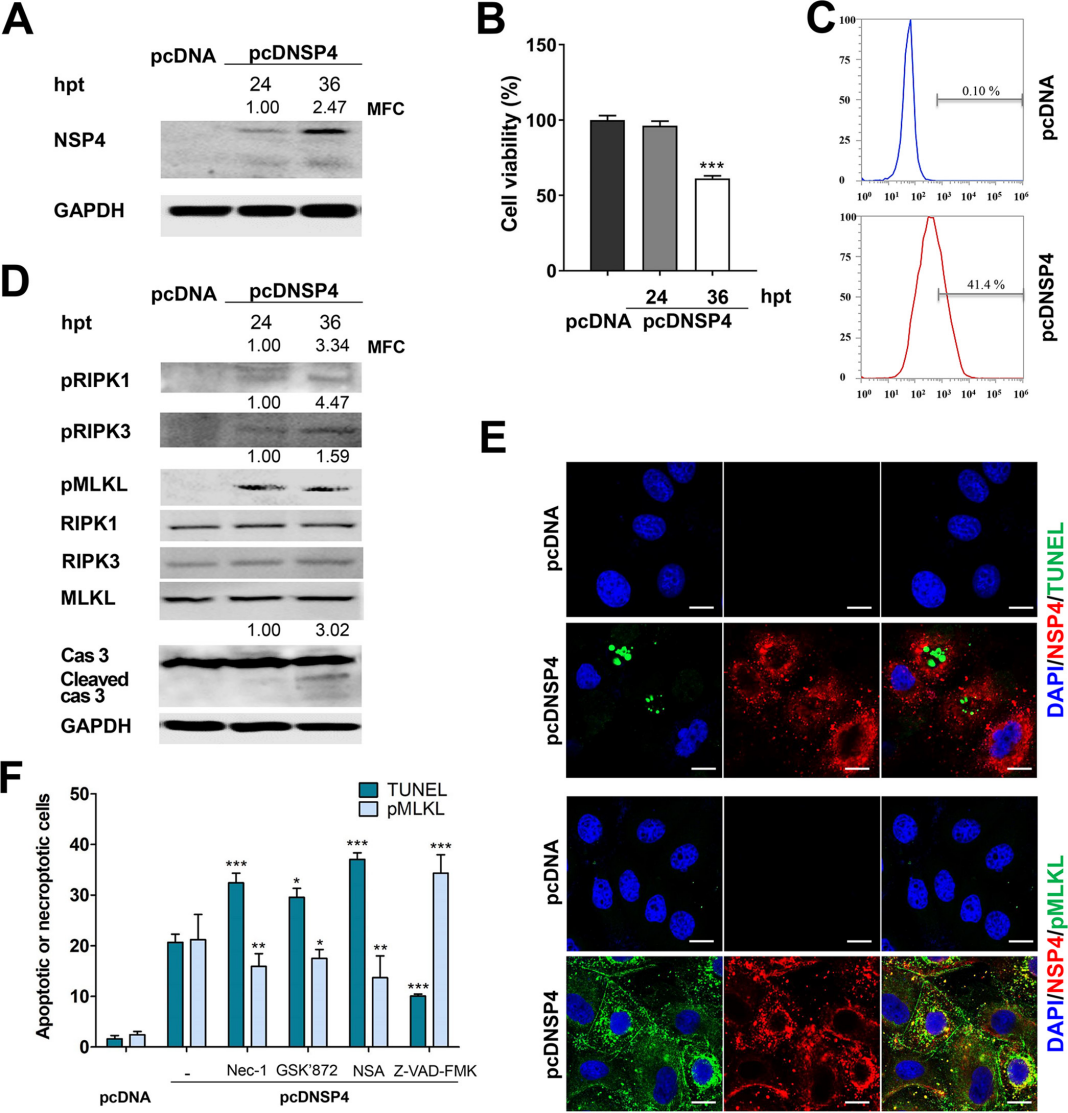
Induction of both apoptosis and necroptosis by the RVA NSP4 protein. (A) MA104 cells transfected with the control pcDNA or pcDNSP4 encoding RVA NSP4 (pcDNSP4) were harvested at the indicated time points and subjected to Western blot analysis to check the expression level of NSP4. GAPDH was used as a loading control. The expression of NSP4 relative to GAPDH was determined using densitometric analysis. MFC, mean fold change. (B) The viability of MA104 cells transfected with the control pcDNA or pcDNSP4 was evaluated using WST assay with an ELISA reader (OD_450_). The results are expressed as the mean percentages of viable cells for three independent experiments. (C) The transfected cells under the above condition were stained with a hyperimmune rabbit serum against NSP4, and the transfection efficiency was analyzed by flow cytometry. (D) The cell lysates under the above-described experimental conditions were subjected to Western blotting to evaluate the expression of the indicated proteins. GAPDH was used as the loading control. The expression levels of pRIPK1, pRIPK3, and pMLKL relative to GAPDH were determined using densitometric analysis. (E) MA104 cells transfected with the control pcDNA or pcDNSP4 were processed for determining both the NSP4 protein and apoptotic DNA fragment using IF and TUNEL assays or for determining both the NSP4 protein and pMLKL using IF assays. The nuclei were stained with DAPI. Scale bars = 10 μm. (F) MA104 cells transfected with the control pcDNA or pcDNSP4 were treated with the 30 μM Nec-1 (RIPK1 inhibitor), 10 μM GSK’872 (RIPK3 inhibitor), 10 μM NSA (MLKL inhibitor), or 20 μM Z-VAD-FMK (pan-caspase inhibitor) 6 h after transfection, harvested at 36 h, and analyzed using flow cytometry to quantify the number of apoptotic (using TUNEL staining) or necroptotic (using pMLKL antibody) cells. The data are shown as the percentage of apoptotic or necroptotic cells from the total tested and compared with those in the pcDNSP4-transfected cells without any treatment. All experiments were performed in triplicate, and the data in panels B and F represent the means ± standard errors of the means. Differences were evaluated using one-way ANOVA. *, *P < *0.05; **, *P < *0.001; ***, *P < *0.0001.

The NSP4 protein of RVA induces caspase-dependent intrinsic apoptosis by increasing the levels of cytoplasmic Ca^2+^ and the proapoptotic protein Bax (25). We investigated whether RVA-induced necroptosis is mediated via NSP4. We transfected MA104 cells with control pcDNA or pcDNA encoding NSP4 (pcDNSP4) and determined the expression of NSP4 using Western blotting with a hyperimmune rabbit serum against NSP4. Compared to transfection with the control pcDNA, transfection with pcDNSP4 increased the expression of NSP4 in a time-dependent manner (Fig. 13A). We next performed the MTT assay and flow cytometry to examine the viability of MA104 cells transfected with pcDNA or pcDNSP4. MA104 cells transfected with pcDNSP4 exhibited a significant decrease in viability compared to those transfected with pcDNA (Fig. 13B and C). The expression of pRIPK1, pRIPK3, and pMLKL, the major molecules in the RIPK1-dependent necroptosis pathway, increased in the pcDNSP4-transfected MA104 cells (Fig. 13D), and as expected, cleaved caspase-3 was detected in the pcDNSP4-transfected MA104 cells (Fig. 13D). Using confocal microscopy, we visualized the apoptosis and necroptosis of NSP4-positive cells through double staining of NSP4 and apoptotic DNA fragment using TUNEL and IF assays, respectively, or NSP4 and pMLKL using IF. The pcDNSP4-transfected MA104 cells exhibited either apoptotic or necroptotic characteristics (compared to the control pcDNA-transfected cells), and NSP4-positive cells exhibited the presence of TUNEL-positive apoptotic bodies and translocation of pMLKL to the peripheral plasma membrane (Fig. 13E). Evaluation of apoptotic and necroptotic cells in pcDNSP4-transfected MA104 cells using flow cytometry revealed that the number of apoptotic and necroptotic cells increased significantly compared with that observed in cells transfected with pcDNA (Fig. 13F). Moreover, the proportion of apoptotic cells increased after the inhibition of necroptosis by treatment with RIPK1, RIPK3, and MLKL inhibitors and vice versa after treatment with the apoptosis inhibitor (Fig. 13F). These results indicate that NSP4 induced apoptosis as well as necroptosis, and as in case of the above-mentioned results, they work in contrasting manners.

## DISCUSSION

Viral infection usually culminates in cell death, and apoptosis is the most extensively studied type of cell death (3, 6, 17, 58). Recently, necroptosis has been reported to occur in response to infections with several viruses, including reovirus (41, 59), human coronavirus OC43 (48), herpes simplex virus 1 (HSV-1) (16, 47), HSV-2 (47), murine cytomegalovirus (51), influenza A virus (54, 55, 60), and vaccinia virus (44, 61). Studies on RVA-induced cell death modalities have been focused on apoptosis but not on necroptosis. In the present study, RVA infection or NSP4 transfection induced the activation of the three key intermediaries of necroptosis, i.e., RIPK1, RIPK3, and MLKL, and this activation was reduced upon treatment with specific inhibitors. Immunoprecipitation with a RIPK1-specific antibody revealed that RIPK1 formed a complex with RIPK3 and MLKL. Moreover, RVA-infected or NSP4-transfected MA104 cells exhibited translocation of pMLKL to the plasma membrane, a characteristic feature of necroptosis. Thus, these results indicate that RVA infection or NSP4 transfection induces RIPK1-dependent necroptosis.

Apoptosis and necroptosis are generally thought to serve as mechanisms involved in protecting the host against viral infection that help control or restrict viral replication, thereby protecting the entire organism (3, 6, 48, 58). In this study, interestingly, inhibition or silencing of each key necroptotic molecule (RIPK1, RIPK3, or MLKL) in RVA-infected cells using specific chemicals or siRNA significantly reduced the replication of RVA, suggesting that RVA-induced necroptosis is proviral. As shown in this study, indeed, the plasma membrane of virus-induced necroptotic cells is ruptured in response to the translocation of MLKL to the periphery of the plasma membrane, and consequently, all cellular compounds (known as damage-associated molecular patterns [DAMPs]) and viral components are released from the lytic cells (1, 62, 63). This may provide progeny viruses with a chance to spread to other sites or organs and be secreted outside the body. However, under *in vivo* conditions, the lytic nature of necroptosis would trigger an inflammatory reaction via DAMPs and promote the recruitment of white blood cells that digest and liquefy the necrotic tissue and clear pathogens such as viruses (62, 64, 65). Because the consequences of RVA-induced necroptosis *in vitro* may be different from those *in vivo*, we are currently studying whether RVA-induced necroptosis has proviral or antiviral effects *in vivo* using RIPK1^−/−^, RIPK3^−/−^, and MLKL^−/−^ knockout mice.

Virus-induced apoptosis results in the formation of apoptotic bodies containing intracellular organelles, other cytosolic components, and premature and mature viral particles. Apoptotic bodies express new ligands for binding and are subsequently engulfed by phagocytic cells, usually preventing the release of infectious progeny viruses to the outside of cells *in vitro* and *in vivo* (5, 66). In the present study, inhibition of apoptosis using the pan-caspase inhibitor (Z-VAD-FMK) increased RVA replication, indicating that RVA-induced apoptosis is a host defense mechanism. This result is in agreement with the findings of studies in which the inhibition of TMEV- and HIV-1-induced apoptosis of infected murine macrophages and CD4 T lymphocytes, respectively, induced a transition from apoptosis to necroptosis and resulted in significantly higher viral titers (49, 50). Taken together, our data suggest that the RVA-induced RIPK1-dependent pathway acts as a proviral mechanism that promotes viral replication and/or spread, whereas—consistent with the findings of a previous study (56)—RVA-induced apoptosis is an antiviral strategy that restricts viral replication.

Blocking of RIPK1, RIPK3, or MLKL resulted in increased cell viability as well as apoptosis. The switch from necroptosis to apoptosis—induced in response to treatment with each inhibitor of the necroptosis molecules—could prevent the assembly and release of RVAs from apoptotic cells by the mechanism as described above, thereby reducing the spread of the virus to neighboring cells (3, 5, 66). Consistent with our results, blocking of HIV-1-induced necroptosis with the RIPK1 inhibitor, Nec-1, increased cell viability and the number of apoptotic cells while inhibiting the formation of syncytia (49). In another *in vitro* experiment, silencing of RIPK1 or MLKL in the background of tunicamycin—which can induce necroptosis as well as apoptosis (57, 67)—treatment of L929 cells, a murine fibrosarcoma cell line, triggered a switch from necroptosis to apoptosis (57). Thus, cell viability and death modalities vary depending on the cellular context and type of stimulus. Nevertheless, our data indicate that the RVA-induced RIPK1-dependent pathway acts as a proviral modality in RVA-infected cultured cells by hastening the spread and transmission of virus progeny to the surrounding uninfected cells.

The NSP4 protein of RVA is known to induce apoptosis in infected cells by increasing the level of cytoplasmic Ca^2+^ and phosphorylation of calcium/calmodulin-stimulated protein kinase II (CaMKII), resulting in the activation of the proapoptotic protein Bax (31, 33, 34, 68). In this study, we confirmed induction of apoptosis after transfection of MA104 cells with NSP4 from bovine RVA strain NCDV. Most interestingly, we found that transfection with NSP4 also induced necroptosis via the activation of the RIPK1/RIPK3/MLKL necroptosis pathway. Moreover, NSP4-transfected MA104 cells exhibited a characteristic translocation of pMLKL to the plasma membrane to puncture the cell membrane. Consistent with our results, it was reported that Sendai virus induces RIPK1-dependent necroptosis in human neuroblastoma cells by increasing the cytoplasmic Ca^2+^ concentration and phosphorylation of CaMKII (69). Furthermore, RVA infection is known to induce ER stress and unfolded protein response in infected cells, resulting in RVA-induced apoptosis (70–72). Indeed, the induction of ER stress in response to infection with several viruses results in apoptosis (73–79) as well as necroptosis (57, 80). Taken together, our data suggest that the NSP4 protein of RVA could induce apoptosis as well as necroptosis by increasing the cytoplasmic Ca^2+^ levels and then induce CaMKII phosphorylation and ER stress. Consistent with the case in RVA-infected cells, inhibitors against the key intermediaries of the necroptosis pathway (RIPK1, RIPK3, and MLKL) decreased NSP4-induced necroptosis and induced a transition from necroptosis to apoptosis (along with an increase in cell viability). This indicates that NSP4 regulates apoptosis and necroptosis in RVA-infected cells.

Apoptosis and necroptosis could be simultaneously induced in virus-infected cells via some factors, such as tumor necrosis factor alpha (TNF-α)—as a ligand for its death signaling TNF receptor type 1 (TNFR1)—and dsRNA—as a ligand for RIG-I-like receptors (RLRs) (2, 11, 41, 44, 49, 52, 59). Indeed, induction of TNF-α expression through TLR3 has been observed in cells transfected with RVA dsRNA (81) and in patients with RVA diarrhea (82). Consistently, it has been reported that sensing of the genomic RNA within incoming reovirus type 3 Dearing stain particles through RLRs culminates in necroptosis (39). Therefore, RVA infection is highly likely to induce necroptosis via the activation of TLR3 and RLRs. Interestingly, RVA infection and transfection of NSP4 can induce apoptosis and necroptosis, respectively. However, the exact mechanisms by which RVA infection triggers different cell mortalities remain unclear. Ligand binding-specific receptors or sensors on the cell surface or inside cells recruit multiple proteins called complex I, providing a platform for gathering downstream kinases and effector proteins (83). The internalization of ligand-bound receptors such as TNFR1 confers formation of complex II (apoptosome), which contains deubiquitinated RIPK1, caspase-8, and the adaptor proteins TNFR-1-associated DEATH domain (TRADD) and FAS-associated death domain (FADD) (3, 39, 81–83). The necroptotic signaling complex, called complex IIb or necrosome, is associated with TRADD, FADD, and caspase-8 (83). In complex IIb, depletion of FADD or caspase-8, inhibition of caspase-8, or induction or overexpression of RIPK3 can initiate necroptosis of TNF-treated cells, among which inhibition or deletion of caspase-8 inhibition is mainly responsible (3, 39, 81–83). In contrast, deletion or inhibition of cellular FADD-like interleukin 1β (IL-1β)-converting enzyme inhibitory protein (cFLIP), which inactivates caspase-8 through formation of a heterodimer, induce apoptosis (3, 39, 81–83). Whether factors playing central roles in determining apoptosis and necroptosis as mentioned above are activated or inhibited in RVA-induced apoptotic and necroptotic cells has not been studied in detail. Moreover, the mechanisms modulating these factors in cells infected with RVA and other viruses remain largely unknown. Identifying the detailed mechanisms by which different modalities of cell death are induced by RVA infection forms the basis of our ongoing work.

**FIG 14:**
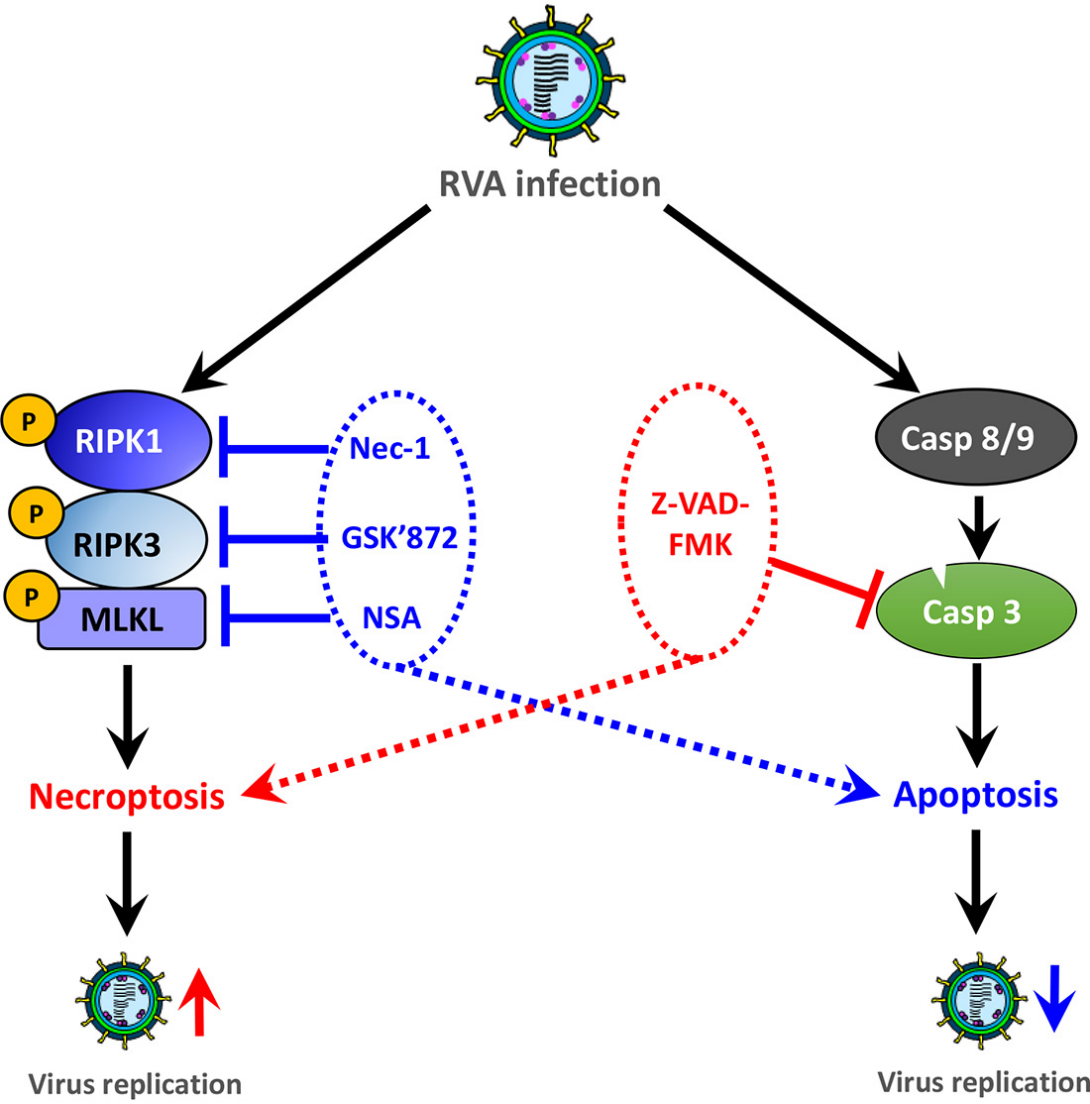
Schematic summary of RVA-induced cell mortality. *In vitro* RVA infection induces both proviral necroptosis and antiviral apoptosis. Inhibition of necrosome molecules, RIPK1, RIPK3, and MLKL, by treatment with chemicals (RIPK1 inhibitor Nec-1, RIPK3 inhibitor GSK’872, MLKL inhibitor NSA) or transfection with each corresponding siRNA converts RVA-induced necroptotic cells partially to apoptotic cells, resulting in suppression of viral replication. In contrast, suppression of RVA-induced apoptosis by treatment with pan-caspase inhibitor (Z-VAD-FMK) switches apoptotic cells partially to necroptotic cells, leading to an increase in viral replication.

In conclusion, our study demonstrates the ability of RVA infection and its NSP4 protein to induce RIPK1-dependent necroptosis in virus-infected cells. Moreover, we identified that RVA-induced necroptosis and apoptosis act as proviral and antiviral mechanisms, respectively (Fig. 14). Our data contribute to the understanding of RVA-induced cell death modalities. Further understanding of the precise molecular mechanism(s) whereby RVA infection induces necroptosis and apoptosis will provide important insights into the development of novel therapeutic strategies against infection with RVA and other RNA viruses.

## MATERIALS AND METHODS

### Cell culture and virus.

Monkey kidney MA104 and human intestinal Caco-2 cells were purchased from the American Type Culture Collection (ATCC; Manassas, VA) and grown in alpha minimal essential medium (α-MEM) and Dulbecco’s modified Eagle’s medium (DMEM; Welgene, Daegu, South Korea), respectively, supplemented with 10% fetal bovine serum, 100 U/mL penicillin, and 100 μg/mL streptomycin. Bovine RVA NCDV (G6P6[1]) and human RVA DS-1 (G2P1B[4]) strains were obtained from the ATCC and propagated in MA104 cells after preactivation with 10 μg/mL porcine trypsin (Gibco, Fort Worth, TX) as described previously (84). Viral titers were determined via immunofluorescence (IF) of infected cells using a monoclonal antibody (MAb) against the VP6 protein of RVA and were expressed as fluorescence focus units (FFU) per milliliter. In all experiments, a multiplicity of infection (MOI) of 1 was used.

### Reagents and antibodies.

Nec-1, GSK’872, NSA, and Z-VAD-FMK were purchased from Calbiochem (Darmstadt, Germany) and dissolved in dimethyl sulfoxide (DMSO) to prepare the stock solutions. SlowFade gold antifade reagent with 4′,6-diamidino-2-phenylindole (DAPI) was obtained from Molecular Probes (Bedford, MA). Mouse MAb against RIPK1, rabbit MAb against MLKL, and rabbit polyclonal antibodies against RIPK3 and pRIPK3 were purchased from Abcam (Cambridge, UK). Rabbit MAbs against pRIPK1, pMLKL, and caspase-3 were obtained from Cell Signaling (Beverly, MA). Rabbit anti-glyceraldehyde 3-phosphate dehydrogenase (anti-GAPDH, FL*–*335) polyclonal antibody was purchased from Santa Cruz (Dallas, TX). Mouse anti-RVA VP6 MAb was obtained from Median Diagnostic (Chuncheon, South Korea). Hyperimmune rabbit serum raised against RVA NSP4 was produced in-house as described below. Secondary antibodies included horseradish peroxidase (HRP)-conjugated goat anti-rabbit immunoglobulin G (IgG) antibody (Cell Signaling), HRP-conjugated goat anti-mouse IgG antibody (AbFrontier, Seoul, South Korea), Alexa Fluor 594 (AF594)-conjugated donkey anti-rabbit IgG antibody, AF594-conjugated goat anti-mouse IgG antibody, and AF647-conjugated goat anti-mouse IgG antibody from Life Technologies (Eugene, OR).

### MTT assay and WST assay.

The cytotoxicity of the chemicals used in this study and their effect on cell viability following RVA infection were determined using the 3-(4,5-dimethylthiazol-2-yl)-2,5-diphenyl tetrazolium bromide (MTT) assay as described elsewhere (85, 86). Briefly, cells grown in 96-well plates were incubated with a medium containing different concentrations of each chemical inhibitor for 24 h. For the cell viability assay, cells grown in 96-well plates were inoculated with RVA strain NCDV or DS-1 at an MOI of 1, treated or not with each chemical inhibitor in a dose-dependent manner, and incubated for the indicated times. After removing the media, 200 μL MTT solution was added to each well and the plates were incubated for 4 h at 37°C in a CO_2_ incubator. Afterward, 150 μL of DMSO was added to each well and the plate was incubated at 20°C for 10 min. The optical density (OD) was read using an enzyme-linked immunosorbent assay (ELISA) reader at 570 nm. Cell viability following transfection of pcDNA or pcDNA encoding NSP4 was determined using the water-soluble tetrazolium salt (WST) assay (CellVia kit; AbFrontier, Seoul, South Korea). Briefly, cells grown in 96-well plates were transfected with the plasmids and incubated for the indicated times. Ten microliters of the WST CellVia reagent was added to each well and the plates were incubated for 4 h at 37°C in a CO_2_ incubator. The OD was read using an ELISA reader at 450 nm. Percent cell viability was calculated using the following formula: [OD_(sample)_ – OD_(blank)_/(OD_(control)_ – OD_(blank)_] × 100. Noncytotoxic concentrations of each chemical were used in this study.

### Treatment of MA104 cells with chemical inhibitors.

MA104 cells grown in 6-, 12-, or 96-well plates were washed twice with phosphate-buffered saline (PBS; pH 7.4) and incubated with either 10 μg/mL porcine trypsin or preactivated RVA NCDV or DS-1 (MOI = 1) at 37°C for 1 h. After washing the cells twice with PBS, the chemical inhibitors RIPK1 (Nec-1; 30 μM), RIPK3 (GSK’872; 10 μM), MLKL (NSA; 10 μM), and pan-caspase (Z-VAD-FMK; 20 μM) or a DMSO vehicle was added to the culture medium for the indicated times as described elsewhere (39, 41, 48, 59).

### Construction and transfection of NSP4 plasmid.

Full-length NSP4 was cloned into pcDNA3.1 as described previously (33, 87). Briefly, cDNA encoding NSP4 was amplified from the NCDV strain (GenBank accession number X06806.1) using the following primers and cloned into the pcDNA6 plasmid: forward, 5′-CACAGGATCCATGGAAAAGCTTACCGACC-3′, and reverse, 5′-AATCTCGAGTTACATCGCTGCAGTCACT-3′, containing BamHI and XhoI restriction sites (underlined), respectively. Transfection of the control pcDNA or pcDNA encoding NSP4 (2.5 μg) was performed in 70 to 80% confluent MA104 cells grown in 12- or 96-well culture plates or 8-well chamber slides using Lipofectamine 3000 (Invitrogen, Carlsbad, CA) according to the manufacturer’s instructions. The cells were harvested at different posttransfection time points and subjected to Western blotting, flow cytometry, and IF assays, as described below.

### Production of rabbit hyperimmune antiserum.

Hyperimmune antiserum against RVA NSP4 was generated as described elsewhere (88, 89). Briefly, cDNA encoding the complete NSP4 genomic segment of strain NCDV was cloned into the expression vector pET28a using the same primers as mentioned above. The NSP4-containing plasmids were transformed to Escherichia coli BL21. Protein expression was induced with isopropyl-β-d-thiogalactopyranoside (IPTG) (1 mM) at room temperature overnight. The His-tagged NSP4 protein was purified using nickel-nitrilotriacetic acid (Ni-NTA) agarose (Qiagen, Valencia, CA), and the protein was eluted with 500 mM imidazole. The concentration of the purified RVA NSP4 protein was determined by measuring the absorbance at 280 nm. Afterwards, two rabbits were subcutaneously immunized with purified NSP4 protein in complete Freund’s adjuvant followed by two booster injections in incomplete Freund’s adjuvant. The animals were bled 2 weeks after the last booster injection. The sera were collected and stored at −20°C until use.

### Knockdown of molecules involved in necroptosis.

MA104 cells were cultured in 12-well culture plates to 70 to 80% confluency and transfected with scrambled siRNA or siRNAs against RIPK1, RIPK3, or MLKL (80 pmol each; Santa Cruz) using Lipofectamine 3000 (Invitrogen) according to the manufacturer’s instructions. To optimize the knockdown efficiency of each siRNA, a second transfection was conducted 24 h after the first transfection. Cells treated in parallel were analyzed using Western blotting to ensure the effective knockdown of each target protein. After confirming the effective knockdown, the cells were infected with preactivated RVA NCDV or DS-1 (MOI = 1). Viral inocula were removed 1 h after infection, and the cells were washed and incubated in α-MEM supplemented with 1 μg/mL porcine trypsin. The cells were harvested 16 h after infection; viral protein expression and viral titer were measured using Western blotting and median tissue culture infective dose (TCID_50_) analyses, as described below.

### Western blotting.

For determining the expression of viral VP6 and target cellular proteins, antibodies specific for each protein were used. Briefly, MA104 or Caco-2 cells grown in 6- or 12-well plates, infected or not with RVA NCDV or DS-1 and treated or not with chemical inhibitors, or MA104 cells transfected with plasmids or siRNAs were washed three times with cold PBS and lysed using a cell extraction buffer (10 mM Tris-HCl [pH 7.4], 100 mM NaCl, 1 mM EDTA, 1 mM EGTA, 1 mM NaF, 20 mM Na_2_P_2_O_7_, 2 mM Na_3_VO_4_, 1% Triton X-100, 10% glycerol, 0.1% SDS, and 0.5% deoxycholate [Invitrogen]) supplemented with 1× concentrations of protease and phosphatase inhibitors (Roche, Basel, Switzerland) for 30 min on ice. The lysates were spun down using centrifugation at 12,000 × *g* for 10 min at 4°C. The supernatants were analyzed for total protein content using a bicinchoninic acid protein assay kit (Thermo Scientific, Waltham, MA). The samples were resolved using sodium dodecyl sulfate-polyacrylamide gel (SDS-PAGE) and transferred onto nitrocellulose membranes (GE Healthcare Life Sciences, Chicago, IL). The membranes were blocked for 1 h at 20°C with Tris-buffered saline containing 5% skimmed milk before overnight incubation at 4°C with the indicated primary antibodies. Then the membranes were incubated with HRP-labeled secondary antibodies and the protein bands were detected using enhanced chemiluminescence (Dogen, Seoul, South Korea) and the Davinch-K imaging system (Youngwha Scientific Co., Ltd., Seoul, South Korea). Protein band intensity was calculated using TINA 2.0 software (Raytest, Staubenhardt, Germany). All data are representative of those from three independent experiments.

### Flow cytometry assay.

To determine the percentage of apoptotic and necroptotic cells, flow cytometry was performed as described elsewhere (31, 90, 91), with slight modification. Briefly, after viral infection and chemical treatment or plasmid transfection, the MA104 cells were washed twice with PBS and detached via incubation at 37°C for 5 min in an Accutase cell detachment solution (BD Biosciences, San Jose, CA). The harvested cells were washed with PBS and pelleted. The cells were divided into two groups for analysis of necroptosis or apoptosis in whole cells, fixed in ice-cold 4% paraformaldehyde for 15 min on ice, permeabilized with 0.2% Triton X-100 for 5 min at 20°C, and then washed twice with PBS. For necroptosis analysis, the cells were incubated with rabbit pMLKL MAb (diluted 1:200 in PBS) at 37°C for 1 h, followed by incubation with AF488-conjugated donkey anti-rabbit IgG antibody (1:200 dilution) at 37°C for 1 h. For apoptosis analysis, the cells were incubated with 50 μL terminal deoxynucleotidyltransferase-mediated dUTP-biotin nick end-labeling (TUNEL) reaction mixture containing fluorescein isothiocyanate (FITC)-conjugated dUTP (*in situ* cell death detection kit; Roche, Mannheim, Germany) for 60 min at 37°C in the dark.

For viral labeling, the cells were incubated with mouse MAb against RVA VP6 (1:200 dilution) at 37°C for 1 h, followed by incubation with AF647-conjugated goat anti-mouse IgG antibody (1:200 dilution) at 37°C for 1 h. For evaluating dually positive cells, RVA, and necroptotic or apoptotic cells, mock- or RVA-infected cells were incubated with an antibody against RVA VP6 along with necroptotic or apoptotic markers at 37°C for 1 h. The stained cells were washed, resuspended in 500 μL cold PBS, and analyzed directly using an Attune NxT flow cytometer (Thermo Scientific), and the data from each sample were digitized using Attune NxT software v3.1.2.

### TUNEL assay.

To determine the percentage of apoptotic cells, the TUNEL assay was performed as described elsewhere (39, 90). TUNEL staining for fluorescence-activated cell sorting (FACS) analysis was performed using an *in situ* cell death detection kit (Roche) according to the manufacturer’s protocol. Briefly, MA104 cells were mock inoculated or inoculated with the NCDV strain and then harvested at different time points. For TUNEL labeling, cells harvested, fixed, and permeabilized as described above were labeled with the TUNEL reaction mixture (1:9 ratio of enzyme solution and label solution) for 1 h at 37°C in the dark. Negative controls were created by omitting terminal deoxynucleotidyl enzyme and adding the same volume of the label solution. The cells were analyzed using an Attune NxT flow cytometer (Thermo Scientific).

### IF.

IF was performed to visualize necroptotic or apoptotic cells as described before (14, 31). MA104 cells grown in 8-well chamber slides were treated or not with trypsin, infected or not with trypsin-activated-RVA strain NCDV, or transfected with plasmids for the indicated times. The cells were then fixed in 4% paraformaldehyde for 15 min, permeabilized with 0.2% Triton X-100 for 10 min at 20°C, and rinsed with PBS. The cells were then incubated with the TUNEL reaction mixture containing FITC-conjugated dUTP for apoptotic cells or rabbit pMLKL MAb for necroptotic cells. For costaining with viral antigen, the cells were incubated with a mouse MAb against rotavirus VP6 with the necroptotic marker or apoptotic TUNEL reaction mixture. After washing with PBS, the cells were incubated with the relevant secondary antibodies and mounted using SlowFade gold antifade reagent containing 1× DAPI (Molecular Probes) for nuclear staining. The infected cells were observed with an LSM 510 confocal microscope and analyzed using LSM software (Carl Zeiss). The numbers of apoptotic and necroptotic cells were quantified by counting 100 cells from each sample and expressed as the percentage of the total population.

### Immunoprecipitation assay.

Immunoprecipitation of each target protein was performed as described previously (92). MA104 cells grown in 6-well plates were mock infected or infected with the RVA NCDV or DS-1 strain at an MOI of 1 and incubated for the indicated times at 37°C. The cell lysates were precleared by incubation with protein A- or G-agarose beads (Santa Cruz) for 30 min at 4°C. The precleared cell lysates were incubated overnight with an antibody against pRIPK1 at 4°C. The immune complexes were captured by incubating with protein A-agarose beads for 1 h at 4°C, and the immunoprecipitated proteins were then evaluated by Western blotting as described above.

### Virus titration using the TCID_50_ assay.

MA104 cells grown in 6-well plates were infected or not with RVA NCDV or DS-1 (MOI = 1) and treated or not with chemical inhibitors. After three freeze-thaw cycles, 10-fold serial dilutions of clarified virus supernatants were prepared in 10 μg/mL CT, inoculated into monolayers of MA104 cells cultured in 96-well plates, and incubated at 37°C. The viral titers were determined at 24 hpi and expressed as TCID_50_ per milliliter using the method proposed by Reed and Muench (93).

### Statistical analysis.

All statistical analyses were performed on triplicate experiments using one-way analysis of variance (ANOVA) and the GraphPad Prism software version 5.03 (GraphPad Software Inc., La Jolla, CA). *P* values of less than 0.05 were considered significant.
